# Sm-p80-based schistosomiasis vaccine mediated epistatic interactions identified potential immune signatures for vaccine efficacy in mice and baboons

**DOI:** 10.1371/journal.pone.0171677

**Published:** 2017-02-13

**Authors:** Juan U. Rojo, Michael W. Melkus, Kameswara Rao Kottapalli, Oscar E. Okiya, Justin Sudduth, Weidong Zhang, Adebayo J. Molehin, Darrick Carter, Afzal A. Siddiqui

**Affiliations:** 1 Center for Tropical Medicine and Infectious Diseases, Texas Tech University School of Medicine, Lubbock, Texas, United States of America; 2 Center for Tropical Medicine and Infectious Diseases, Texas Tech University School of Medicine, Amarillo, Texas, United States of America; 3 Center for Biotechnology and Genomics. Texas Tech University, Lubbock, Texas, United States of America; 4 Department of Internal Medicine. Texas Tech University School of Medicine, Lubbock Texas, United States of America; 5 PAI Life Sciences, Seattle, Washington, United States of America; 6 Infectious Disease Research Institute, Seattle, Washington, United States of America; 7 Department of Global Health, University of Washington, Seattle, Washington, United States of America; University of Illinois, UNITED STATES

## Abstract

Schistosomiasis is a neglected parasitic disease of major public health concern as it affects over 250 million people in developing countries. Currently there is no licensed vaccine available against schistosomiasis. The *Schistosoma mansoni* calpain protein, Sm-p80, is a leading vaccine candidate now ready to move to clinical trials. In order to better assess Sm-p80 vaccine immunogenicity; here we used a systems biology approach employing RNA-sequencing to identify gene signatures and epistatic interactions following Sm-p80 vaccination in mouse and baboon models that may predict vaccine efficacy. Recombinant Sm-p80 + CpG-oligodeoxynucleotide (ODN) vaccine formulation induced both cellular and humoral immunity genes with a predominant TH1 response as well as TH2 and TH17 gene signatures. Early gene responses and gene-network interactions in mice immunized with rSm-p80 + ODN appear to be initiated through *TLR4* signaling. *CSF* genes, *S100A* alarmin genes and *TNFRSF* genes appear to be a signature of vaccine immunogenicity/efficacy as identified by their participation in gene network interactions in both mice and baboons. These gene families may provide a basis for predicting desirable outcomes for vaccines against schistosomiasis leading to a better understanding of the immune system response to vaccination.

## Introduction

Vaccines are one of the most cost-effective approaches to control infectious diseases [1–3]. The parasitic disease schistosomiasis currently affects over 250 million people with a global impact on over a billion people in more than 78 countries [4]. Despite integrated control measures, schistosomiasis continues to spread to new regions [5]. Therefore, the development of a protective vaccine against schistosomiasis still remains potentially the most effective means for controlling the disease [6, 7]. *Schistosoma mansoni* calpain (Sm-p80) has been shown to elicit immune protection against schistosomiasis making it a promising vaccine candidate and is now in preparation for GMP production for Phase I/II human clinical trials [8–10]. In order to further evaluate and understand vaccine-mediated mechanisms of protection, it is important to demonstrate their effectiveness and identify correlates of protection. Recently, systems vaccinology has been successfully applied in dissecting the molecular mechanism of human approved vaccines for yellow fever, influenza, and meningococcus [11–13]. However, this approach has not been employed using next-generation sequencing (NGS) in the evaluation of a non-licensed parasitic vaccine. In this study, we employed NGS to understand epistatic interactions that correlate to Sm-p80 vaccine immunogenicity and disease protection in mouse and baboon models of infection. In order to do so, we integrated data obtained from different RNA-sequencing experiments. First, we identified transcriptional responses to five different Sm-p80 vaccine formulations in mice. Second, we identified early-gene immune responses to rSm-p80 + ODN vaccine formulation in mice. Third, we compared mice gene responses to Sm-p80 vaccination and *S*. *mansoni* infection in naïve mice. Last, we identified epistatic interactions in response to Sm-p80 vaccination in baboons across different immune system tissues, peripheral blood mononuclear cells, spleen, and lymph nodes. Our results describe the potential of RNA-sequencing to identify gene interactions as possible correlates of vaccine mediated protection in mice and baboons.

## Materials and methods

### Ethics statement

This study was carried out in strict adherence with the recommendations for the Care and Use of Laboratory Animals of the National Institutes of Health. Naïve female 4–6 weeks old C57BL/6 mice were purchased from Charles River Laboratories International Inc. (Wilmington, MA, USA). The Institutional Animal Care and Use Committee (IACUC) of Texas Tech University Health Sciences Center Laboratory Animal Resource Center approved the use of mice for this study. Male and female non-human primates, olive baboons (*Papio anubis*) ages 2.3 to 6.29 years old were obtained from the University of Oklahoma Health Sciences Center (OUHSC; Oklahoma City, OK) baboon breeding colony and housed in Association for Assessment and Accreditation of Laboratory Animal Care accredited facilities at OUSHC.

Mice studies presented in this publication were approved by the IACUC (Protocol Number 20010202 (Texas Tech University Health Sciences Center). Animals are housed in facilities maintained by the animal resources program at the TTUHSC. The Animal Research Facilities meet the NIH standards as set forth in the “Guide for the Care and Use of Laboratory Animals” and accepts as mandatory the PHS “Policy on Humane Care and use of Laboratory Animals by Awardees Institutions” and NIH “Principals for the Utilization and Care of Vertebrate Animals Used in Testing, Research and Training”. TTUHSC (A-3056-01) has on file with the Office for Protection from Research Risk an approved Assurance of Compliance. Mice were be sacrificed by CO_2_ asphyxiation followed by cervical dislocation following the recommendations of the Panel on Euthanasia of the American Veterinary Medical association. Nonhuman primate (baboon) studies presented in this publication were approved by the IACUC (Protocol Number: 11-160-I (Oklahoma University Health Sciences Center). OUHSC maintains a USDA-reviewed program for environmental enhancement to promote psychological well-being of our nonhuman primates. OUHSC also have been an Assured Institution (Category 1, #A-3165-01) in full compliance with the Public Health Service Policy and approved by the Office for Protection from Research Risks since 1986. Social housing, structural enrichment, cage manipulanda, interaction with animal care staff, and novel dietary supplementation are standard procedures for our primate populations as to ensure regular food and water, sufficient space, and social interactions and stimulation for play. To minimize distress caused by the isolation of protocol regimens, baboons are returned to the colony/cage-mate as soon as possible or housed separately but adjacent to familiar cohorts. To minimize distress, baboons were sedated with ketamine 10 mg/kg IM. All animals were euthanized by deeply anesthetizing with sodium pentobarbital. Once a deep surgical level of anesthesia is reached, baboons were exsanguinated via cardiac puncture. Procedures are in accordance with the recommendations of the 2013 Report of the AVMA Panel on Euthanasia. Prior to experiment all baboons were prescreened for intestinal and blood parasites, as well as tested for serum antibody cross-reactivity with Sm-p80. All animals were found negative for parasites and Sm-p80 cross-reactive antibodies. The use of animals for this study was approved by the Institutional Care and Use Committee. *Biomphalaria glabrata* (Puerto Rican strain), snails, were obtained from the National Institute of Allergy and Infectious Diseases Schistosomiasis Resource Center, Biomedical Research Institute (Rockville, MD, USA).

### Sm-p80 vaccine preparations

#### DNA vaccine

Sm-p80-DNA based vaccine formulation protection efficacy in mice has already been reported [14]. Briefly, the large subunit of *S*. *mansoni* calpain, Sm-p80, was cloned into the *Bam*HI site of the mammalian expression plasmid VR1020 (Vical Incorporated, San Diego, CA, USA). Plasmid DNA containing Sm-p80 was obtained by alkaline lysing transformed *E*. *coli* hosting the vector. The resulting DNA was purified on Sepharose CL4B colum and ethanol precipitated for resuspension in sterile, endotoxin-free phosphate buffered saline.

#### Recombinant protein vaccine

Details of the preparation of recombinant Sm-p80 protein vaccine have been described elsewhere [15]. Briefly, the full-length coding sequence of Sm-p80 was cloned into pCold II vector for transformation in *E*. *coli* BL21 (DE3). Transformed cells were cultured in LB plus ampicillin at 37°C and cold induced using IPTG. Harvested cells were re-suspended in pCold lysis buffer for sonication. Cell lysate supernatant was collected and incubated with nickel resin for binding. Imidazole was used for protein elution and dialyzed. Protein expression was confirmed by SDS-PAGE and immunoblotting. Three different rSm-p80 vaccine formulations were tested on different adjuvants: one, coupled with TLR7 agonist alum; two, coupled with TLR9 ligand, CpG-ODN, and three, coupled with TLR4 agonist glucopyranosyl lipid A oil-in-water stable emulsion formulation (GLA-SE). Prior injection, rSm-p80 was added and mixed with the designated adjuvant to make the final vaccine formulation.

#### Heterologous prime/boost vaccine

Sm-p80-based dual vaccine strategy (prime/boost) was a combination of DNA vaccine preparation, pcDNA3-Sm-80, and recombinant protein Sm-p80 in CpG-ODN preparation. Mice were primed with DNA vaccine, and then boosted two times at 4 week intervals with rSm-p80 + ODN [15].

### Immunization strategies

#### Sm-p80 vaccine formulations study

Each immunization regimen consisted of 30 mice, 15 animals randomly assigned as control and experimental groups, respectively. For DNA vaccine strategy, experimental mice were immunized with 100μg Sm-p80-VR1020 plasmid DNA on weeks 0, 4, and 8. Control animals were immunized with the same concentration with empty vector VR1020 at the same time-intervals. For prime/boost immunization strategy, experimental animals received 100μg of DNA vaccine (Sm-p80-pcDNA3) with 50μg CpG ODN (101014) in one leg and 25μg of recombinant protein (rSm-p80) plus 50μg CpG ODN in the other leg. Mice in control group received the same dosages of DNA vector with ODN. For recombinant protein immunization in alum, experimental mice were vaccinated with 25μg rSm-p80 in 150μg alum at weeks 0, 4, and 8; control mice received 150μg alum only. For recombinant Sm-p80 immunization in CpG ODN strategy; experimental animals were administered 25μg rSm-p80 in 50μg CpG ODN 101104 at weeks 0, 4, and 8; control mice received 50μg CpG ODN 2137. Lastly, for rSm-p80 plus GLA-SE vaccine strategy; experimental animals were injected with 25μg rSm-p80 in 5μg GLA-SE while control animals received 5μg GLA-SE only at week 0, 4, and 8. All injections were intramuscularly administered [14, 15].

#### Early-gene responses in mice study

Mice were randomly and equally distributed according to given treatment: control (ODN) and experimental (rSm-p80 + ODN) groups, where n = 30 for each group. Five mice were also assigned as pre-treatment control, naïve, for baseline comparisons (n = 5). To study the time-dependent transcriptome response to Sm-p80 vaccination in mice we collected spleens at different time intervals after vaccination: 24 hours, 48 hours, 7 days, and 21 days post-vaccination. At time 0, mice from control and experimental groups were treated. Control mice were administered 50μg of CpG-ODN2137 re-suspended in PBS. Experimental group mice were immunized with a total concentration of 25μg of rSm-p80 and 50μg of CpG-ODN10104 re-suspended in PBS. All immunizations were administered intra-muscularly in the mice’s quadriceps. At 24 hours post-vaccination, five mice from each group were euthanized by carbon dioxide asphyxiation followed by cervical dislocation. Immediately, cardiac puncture was performed for blood collection followed by splenectomy for splenocytes harvest. Likely, at 48 hours, 7 days, and 21 days post-vaccination, mice were euthanized and tissues were collected as described.

#### Gene responses in baboon study

To perform the experiment 8 baboons were randomly and equally designated into control (ODN only) and experimental (rSm-p80 + ODN) groups. Animals were immunized with 250 μg rSm-p80 plus 250 μg ODN. All animals received prime immunization followed by two boosts at 4 week intervals. After the last immunization all animals were percutaneously exposed to 1,000 *S*. *mansoni* cercariae each. To allow for disease progression and egg laying animals were under observation for 8 weeks [9].

### Parasite challenge

#### Mouse model

At week 12-post prime immunization all mice were challenged with *S*. *mansoni* cercariae. Each mouse was infected with 150 cercariae via tail exposure. The 150 cercariae were then pipetted to a glass test tube and filled up with spring water. Each mouse was then inserted into a restrainer tube with its tail placed into the test tube containing the cercariae. After 8 weeks, mice were euthanized by carbon dioxide asphyxiation followed by cervical dislocation. Adult worms are counted from the perfusate and mesenteric veins are inspected to collect any worms remained in tissues. Worm reduction in vaccinated versus control animals was calculated from the number of worms recovered from each mouse (worm burden). Worm burden reduction was then calculated using the standard formula: %P = $\frac{C-I}{C\;x\;100}$; where *P* equals protection, *C* equals average worm burden of control animals, and *I* equals average worm burden of experimental animals.

#### Baboon model

All animals were percutaneously exposed to 1,000 *S*. *mansoni* cercariae each [9]. To allow for disease progression and egg laying animals were under observation for 8 weeks. Following disease progression observation period, animals were euthanized. Adult *S*. *mansoni* worms were recovered by perfusion of the liver ad intestines, followed by careful visual inspection of the intestinal mesenteric veins.

#### Tissue collection

Spleens were mashed with the aid of a syringe plunger and washed with RPMI supplemented with antibiotics (kanamycin, gentamycin, and streptomycin). The resulting cell pellets were re-suspended in freezing media and aliquoted for storage at -80°C.

Baboon blood samples were collected in heparinized vacutainer tubes for storage until processing. Peripheral blood mononuclear cells (PBMCs) were isolated from whole blood using Histopaque-1077 (Sigma Aldrich, St. Louis, MO). Purified PBMCs were re-suspended in freezing media (10% DMSO in fetal bovine sera plus RPMI) for storage and later use. Cell suspensions were washed and stored in freezing media until use.

### Total RNA extraction

Total RNA was extracted and RNA-Seq libraries were prepared from individual samples from each animal. Total RNA was extracted using QIAGEN RNeasy Mini Prep Kit (Redwood City, CA) following manufacturer’s instructions. Briefly, PBMCs, splenocytes, and lymph node cells stored in freezing media were centrifuged to collect cell pellets. Immediately, RLT buffer was added to the pellets for cell lysis and homogenization. Homogenized samples were passed through a genomic DNA shredder column and ethanol was added to adjust binding conditions. Samples were then passed through an RNeasy spin column for RNA absorption and washing. Total RNA was then eluted in DEPC treated water. Total RNA concentrations were calculated using Qubit® 3.0 fluorometer and RNA HS assay kit. RNA samples were stored at -80°C until sequencing library preparation.

### Library preparations and RNA-sequencing

#### Mouse

Complementary DNA libraries for sequencing were prepared from total RNA extracted from individual mouse spleens. From each sample, 3μg of total RNA was used for cDNA library construction following the protocol supplied with the TruSeq® Stranded mRNA HT sample preparation kit (illumina, San Diego, USA). Briefly, total RNA was incubated with magnetic oligo (dT) beads and heat denatured to facilitate binding of the polyA RNA to the beads for mRNA isolation. Beads were then washed to discard rRNA and other contaminants to posteriorly fragment the libraries. Cleaved RNA fragments that were primed with random hexamers were then transcribed into first strand cDNA using SuperScript® III reverse transcriptase (Thermo Fisher Scientific, Waltham, MA, USA) and random primers, followed by second strand cDNA synthesis using DNA polymerase I. The resulting blunt-ended dscDNA were then 3’ adenylated to prevent the fragments from ligating to one another and to provide a complementary overhang for ligating adapters. Libraries were then dual-indexed using unique combination of indexes for each sample. The resulting indexed libraries were enriched using PCR to selectively amplify DNA fragments that have adapter molecules on both ends and to amplify the amount of DNA in the library. The resulting libraries were cleaned using Agencourt AMPure XP purification beads (Beckman Coulter, Brea, CA, USA) and re-suspended in 30μL of resuspension buffer. Library preparation resulted in 56 dual-indexed libraries with a mid-insert size of 300 bp. Libraries were validated for insert size by capillary gel electrophoresis on an Agilent 2200 TapeStation instrument using D1000 ScreenTape for analysis (Santa Clara, CA, USA). All libraries were quantified in triplicates using Qubit® 3.0 fluorometer using dsDNA HS assay kit. Prior to cluster generation for sequencing, samples were normalized to an equimolar concentration. Validated and indexed stock libraries were diluted in 10mM Tris-HCl, pH 8.5 with 0.1% Tween 20 to 10nM, concentrations were quantified in triplicates using Qubit 3.0® fluorometer. The resulting 10nM libraries were then further diluted to 5nM for pooling. The pooled cDNA libraries were denatured, diluted, and loaded in a HiSeq 2500 sequencer (illumina, San Diego, USA). Paired end sequencing with 2X125 bp read length was performed using HiSeq Rapid SBS Kits with v2 chemistry.

#### Baboon

Due to limited RNA concentrations to prepare libraries through conventional kits (illumina TruSeq® kits); cDNA was prepared before library preparation using Smarter kit (Clonetech Laboratories, Inc. Mountview CA). A total of 10pg total RNA was used as input for cDNA synthesis following manufacturer’s protocol described in SMART-Seq v4 Ultra Low Input RNA kit for sequencing. Resulting cDNA was used for sequencing library construction following Nextera® XT DNA library preparation protocol which fragments and tags (tagmentation) dscDNA. The resulting dual-indexed paired-end libraries were validated for insert size Agilent 2200 TapeStation (Agilent Technologies). Library concentrations were measured in triplicates using Qubit® fluorometer and dsDNA HS assay kit. Library normalization was performed to ensure equal library representation in the pooled sample. Libraries were diluted in 10mM Tris-HCl, pH 8.5 with 0.1% Tween 20 to a final concentration of 4nM. A total of two pools were prepared; one pool containing all PBMCs samples, the second pool containing all lymph node and splenocytes samples. After pooling, samples were denatured with 0.2N NaOH and loaded into a cBot for cluster generation in a V4 flow cell. After cluster generation, the flow cells were transferred to an illumina HiSeq for 2 X 125 bp paired end sequencing utilizing v4 chemistry in a high output run mode.

### Bioinformatics

#### Mouse

Sequencing run data containing base call information was demultiplexed in BaseSpace® Sequence Hub cloud-based genomics computing environment for next-generation sequencing (NGS) data management and analysis tool. Samples were analyzed automatically using the illumina workflow apps according to the designed sample sheet. The resulting fastq.gz files were decompressed using 7-zip software to generate fastq files for read 1 and read 2 for each sample. Raw reads quality assessment was performed by QC statistics with FastQC software tool (Babraham Bioinformatics). For raw-read alignment and differential gene expression analysis fastq files were imported into QSeq® Version 13.0 software (DNASTAR, Madison, WI, USA) and aligned to *Mus musculus* reference genome (assembly GRCm38.p4) with reads assigned per kilobase of target per million mapped reads (RPKM) normalization. Student’s two-tailed unpaired t-test with Benjamin Hochberg FDR correction was used to compare the means of gene expression values for five individual replicates for a given gene. RNA-Seq QSeq data was analyzed by creating replicate group sets according to mice post-vaccination time-point and treatment group. The genes were identified as differentially expressed with ≥ two-fold changes if they had the false discovery rate method for multiple test correction *p*-value of ≤ 0.05. For each observation, the resulting differentially expressed genes (DEGs) with annotations, Log_2_ fold-change, and *p*-value were exported for input for into pathway mapping into Ingenuity Pathway Analysis Software (IPA®, QIAGEN Redwood City) tool core analysis using standard settings with duplicates resolved. Comparison analysis was performed to analyze differences across time-points post-vaccination in control and experimental animals. The network molecules associated with an immune system response in the Knowledge Base were considered for analysis and identification of key molecular signatures as well as molecules and pathways with greatest activation z-score difference were selected for further analysis. Right-tailored Fisher’s exact test was used to calculate the *p*-value to reflect the likelihood of an overlap between a set of molecules and a given process is due to random chance. Activation z-score statistic was used to predict the directional effect of one molecule on another or on a process to predict the activation state (inhibition/activation). Heat maps were generated using Gene-E (Broad Institute, https://software.broadinstitute.org/GENE-E/).

#### Baboon

Sequencing run data containing base call information was stored and demultiplexed in BaseSpace® Sequence Hub. The resulting fast.gz files were decompressed using 7-zip to obtain fastq files for read 1 and read 2 for each sample. Raw reads quality assessment was performed by QC statistics with FastQC software tool (Babraham Bioinformatics). A summary of the quality scores for the sequencing run are depicted in S5 Dataset. The resulting folders containing reads 1 and 2 for each animal for each tissue sample were aligned to the *Homo sapiens* Human Genome version 19 downloaded from NCBI RefSeq using QSeq® version 13.0 software (DNASTAR, Madison, WI, USA) for differential gene expression analysis using RPKM normalization. RNA sequencing data was analyzed by creating different replicate groups. Samples were separated by tissue of origin (lymph node, spleen, or PBMCs), experimental group assignation, and, in the case of PBMC samples, separated by time-point. Differential gene expression analysis was performed by comparing the experimental group samples to their corresponding control samples. The resulting DEGs with annotation, log_2_ fold-change, *p*-value, and standard deviation, were exported. Differentially expressed gene lists were then loaded into PANTHER Classification System for gene ontology. Gene list files were also uploaded into Ingenuity Pathway Analysis Software tool core analysis using standard setting with duplicates resolved. IPA analysis consisted of identifying canonical pathways, upstream regulators, molecular networks, and regulator effects associated with an immune system response, cell signaling, and cellular development were considered for identification of key molecules and network mapping. Right-tailored Fisher’s exact test was used to calculate the *p*-value to reflect the likelihood of an overlap between a set of molecules and a given process is due to random chance. Activation z-score statistic was used to predict the directional effect of one molecule on another or on a process to predict the activation state (inhibition/activation).

### Quantitative real-time PCR

Real-time PCR was carried out to determine and validate the expression levels of selected genes as well as to identify rSm-p80 + ODN immunized mice in a blinded study. Specific primers for qRT-PCR were designed from mRNA sequences obtained from NCBI for mice genes: *CSF2*, *TLR4*, *S100A8*, *HEBP1*, *STAT1*, *FOS*, *CDK1*, and *GAPDH*. Selected baboon DEGs were used as reference for template-guided assembly using raw reads to obtain mapped contings. Contig consensus was obtained using N-Gen and SNPs were modified according to general consensus among contigs. Primers were designed and obtained using PrimerQuest from Integrated DNA Technologies (IDT, Coralville, IA). All primer sequences for qRT-PCR are shown in S6 Dataset. The final concentrations of both forward and reverse primers for each reaction were 0.2 μM in a 100μL. Briefly, synthesized cDNA were used as templates for PCR amplification using SYBR Green PCR Master Mix (Takara, Japan) on a StepOne^TM^ Plus Real time PCR system (Applied Biosystems) The reaction mixture was prepared on MicroAmp® 96-well reaction plate. Reaction conditions were: initial denaturation at 95°C for 30 seconds; PCR amplification for 40 cycles at 95°C for 5 seconds and 60°C for 30 seconds. GAPDH was used as the housekeeping gene for normalization. All reactions were carried out in triplicates. Results were analyzed using DataAssist^TM^ software v3.0. Graphpad Prism v7 was used to assess the statistical significance of the fold change in expression pattern (S8 Fig).

### Independent blinded vaccination

Ten mice were equally, randomly, and blindly administered either rSm-p80 + ODN or ODN alone. Twenty-four hours post immunization mice were euthanized and spleens were processed to isolate RNA and synthesize cDNA for qRT-PCR studies of selected genes (*CSF2*, *TLR4*, *S100A8*, *FOS*, *HEBP1*, and *STAT1*). Based on expression levels for each gene mice were assigned to an experimental group. After assessment of all expression levels for all genes the animals were categorized as ODN or rSm-p80 + ODN. All animals were un-blinded to identify the accuracy of the identification.

### Data acquisition

Sequencing quality scores were evaluated using FastQC (S5 Dataset) before mapping into the corresponding genome. Gene expression differences were obtained using DNASTAR-QSeq [16]. DEGs were used as input to generate CIRCOS plots, gene ontology classification was performed using PANTHER GO; networks and pathways were generated using Ingenuity Pathway Analysis (IPA®, QIAGEN Redwood) and Cytoscape [17–20]. Data from each experiment were deposited at NCBI-SRA under Bioprojects: SRP079915, SRP081153, SRP081155, and SRP081154. Individual accession numbers for all samples in each BioProject are described in detail in S1 Dataset.

## Results

### Transcriptomics comparison for five different Sm-p80 vaccine formulations

**Fig 1:**
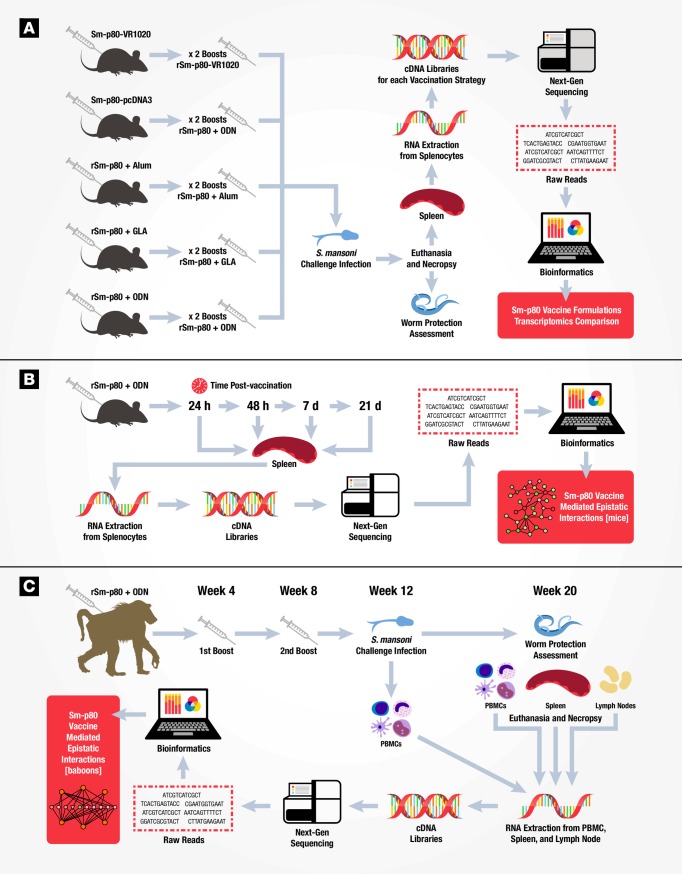
Workflow schematic to evaluate Sm-p80 vaccine efficacy in mouse and baboon models. **(A)** Experimental design to evaluate five Sm-p80 vaccine formulations for protection against *Schistosoma mansoni* challenge using transcriptomics. Thirty mice for each vaccination strategy (n = 15 for control and experimental) were immunized with DNA based vaccine (VR1020-Sm-p80); prime-boost approach (primed with DNA vaccine pcDNA3-Sm-p80 and boosted with recombinant Sm-p80 in ODN adjuvant); or with recombinant protein based vaccine (rSm-p80) in different adjuvants (alum, ODN, or GLA). Vaccinated control animals received empty DNA vector or adjuvant alone. All vaccinated animals received two booster immunizations prior to challenge infection with *S*. *mansoni*. For control challenge mice, naïve animals were infected with *S*. *mansoni* (not shown in figure). Eight weeks post-infection, animals were euthanized and necropsied to assess worm burden protection and tissue collection. Splenocytes from individual mice were pooled for RNA extraction and construction of cDNA libraries for high-throughput sequencing. Bioinformatics and transcriptomics allowed Sm-p80 vaccine formulations comparisons to identify signature molecules and network pathways. **(B)** Experimental design to identify early-gene signatures during the immune response to rSm-p80 + ODN vaccine. Experimental mice (n = 20) were immunized with rSm-80 + ODN while control mice were injected with ODN alone (n = 20). Five animals per group were euthanized at 24 hours, 48 hours, 7 days, and 21 days post-immunization. Splenocyte RNA was isolated and individual mouse cDNA libraries constructed for high-throughput sequencing. Bioinformatics analysis identified gene-networks during the immune response over time to vaccination with rSm-p80 + ODN. **(C)** Experimental design to assess rSm-p80 + ODN vaccine efficacy in a non-human primate model using transcriptomics. Baboons immunized with rSm-p80 + ODN (n = 8) or adjuvant alone (n = 8) received two booster vaccinations at four week intervals each. Prior to *S*. *mansoni* challenge infection (week 12), peripheral blood mononuclear cells were collected to establish a baseline immune signature (distinguish between vaccinated and control). Eight weeks post-infection (week 20) animals were euthanized and necropsied to assess worm burden. Individual tissue samples (PBMCs, spleens, and mesenteric lymph nodes) from each baboon were processed for RNA extraction and library preparation for sequencing. Data analysis identified Sm-p80 induced baboon tissue specific gene networks.

**Fig 2:**
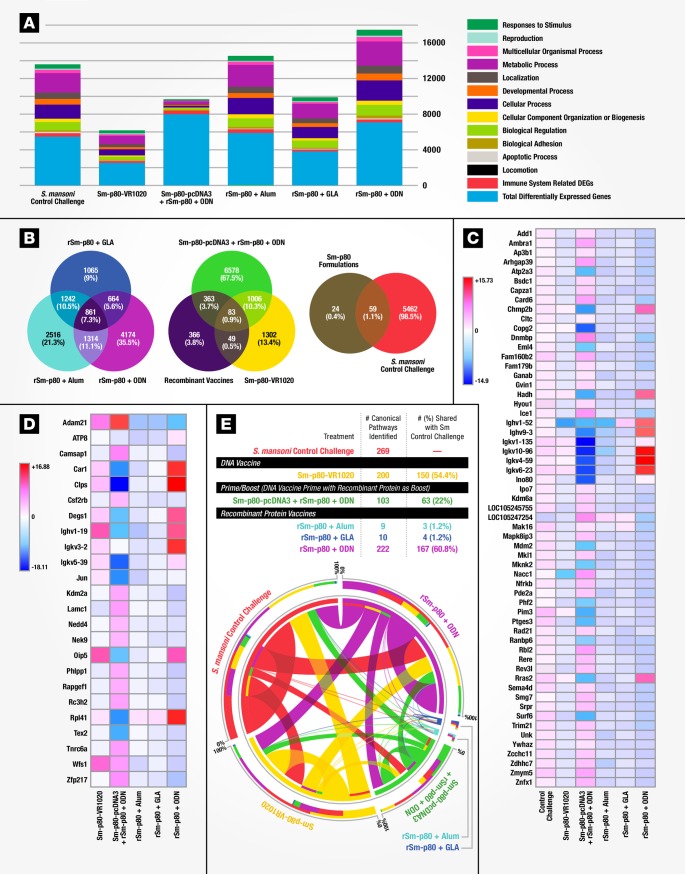
Transcriptomic comparison for five different Sm-p80 vaccine formulations. **(A)** Distribution of differentially expressed mouse splenocyte genes (y axis) according to ontology classification for mice immunized with different Sm-p80 vaccine formulations and challenged with *S*. *mansoni* compared to control challenge in naïve mice (x axis). **(B)** Comparative levels of differentially expressed genes between formulations. **(C)** Heat map for relative gene expression values of 59 common genes (rows) comparing *S*. *mansoni* challenged and Sm-p80 formulations (columns). Identified genes were statistically significant (*P* < 0.01, Student’s *t*-test and greater than 2-fold change with fold change in Log_2_). **(D)**. Heat map showing expression differences of 24 identified genes (rows) unique for Sm-p80 formulations (columns). **(E)** Circular visualization (CIRCOS) plot showing shared canonical pathways across vaccine formulations and control challenged mice. Significant canonical pathways (*P*-value <0.05, right-tailed Fisher Exact Test) were identified and compared across each condition.

We compared three different Sm-p80-based vaccination strategies: A DNA-based vaccine, Sm-p80-VR1020 [14]; Heterologous prime-boost vaccination [15] with Sm-p80-pcDNA3 as a prime boosted with rSm-p80 + the TLR9 agonistic ODN; and the recombinant protein based antigen, rSm-p80, paired with different adjuvants: alum, GLA-SE, or the ODN [21, 22]. For this purpose, mice immunized with a given Sm-p80-based strategy were challenged with *S*. *mansoni* and assessed for worm burden. Here protection levels as measured by reductions in worm burden ranged from 47 to 70%, with rSm-p80 + ODN demonstrating the highest level of protection [14, 15, 21–23]. To explore the transcriptomes and identify gene signatures in which each vaccine induced protective immunity, we used RNA-Seq of mouse splenocytes (Fig 1A). Bioinformatic analysis revealed that in this model rSm-p80 + ODN based vaccines induce the highest number of differentially expressed genes (DEGs) mapping to an immune system process (Fig 2A, S1A Fig and S2 Dataset). Differential expression of a total of 83 genes was common to all vaccination strategies, 59 of which were also induced by *S*. *mansoni* infection in naïve mice while 24 genes were unique to Sm-p80 immunized mice (Fig 2B). Comparison of the 59 genes differentially expressed in both vaccine and infection groups revealed up-regulation of nearly all these genes in the control challenged group while most genes were down-regulated in vaccinated animals. Several immunoglobulin-variable (Ighv and Ighk) region-related genes were observed to be highly upregulated in rSm-p80 + ODN immunized mice, pointing to active Ig-gene rearrangement with a decrease in IghD and an increase in IghA, IghG1, IghG2 gene expression (Fig 2C). Expression levels for the 24 Sm-p80 induced unique genes were found to be more heterogeneous in comparison (Fig 2D). Sm-p80 + ODN differed from the other formulations in that it exhibited characteristics of a TH1 response (*IFNγ*, *IL15*, *IL18*, and *LTBR*), TH2-associated gene expression (*IL4* and *IL13Ra2*) as well as TH17 helper T cell cytokine/receptor expression profiles (*IL17C* and *D* receptors and *IL15*). We also found increased interferon regulatory factors (*IRF3* and *7*) gene expression with many interferon inducible genes upregulated compared to the other vaccine formulations. Other increased signatures included MHC genes (class I and class II); the C1qa/C1qb, C4a/C4b, and C8g components of both the classical and alternative complement pathways; the Ly6 gene family; and—significantly–signaling molecules like Ca^2+^ binding protein *S100A* gene family (alarmins). In addition, we found an increased expression of transcriptional initiation factors, protein folding genes, ribosomal proteins (*Rpl22*—survival of expanding αβ T cells), antioxidant enzymes, proteasome genes important for processing class I peptides and various mitochondrial genes (S1B Fig). Further mapping of DEGs into canonical pathways (S1 Table) identified rSm-p80 + ODN to have 167 canonical pathways in common with the *S*. *mansoni* infection control (Fig 2E). The high similarity of induced canonical pathways between *S*. *mansoni* infected naïve mice and rSm-p80 + ODN immunized mice suggests similar immune transcriptome responses to parasite infection and Sm-p80 vaccination.

### Early gene signatures of immune response to recombinant Sm-p80 + ODN vaccine formulation in mice

**Fig 3:**
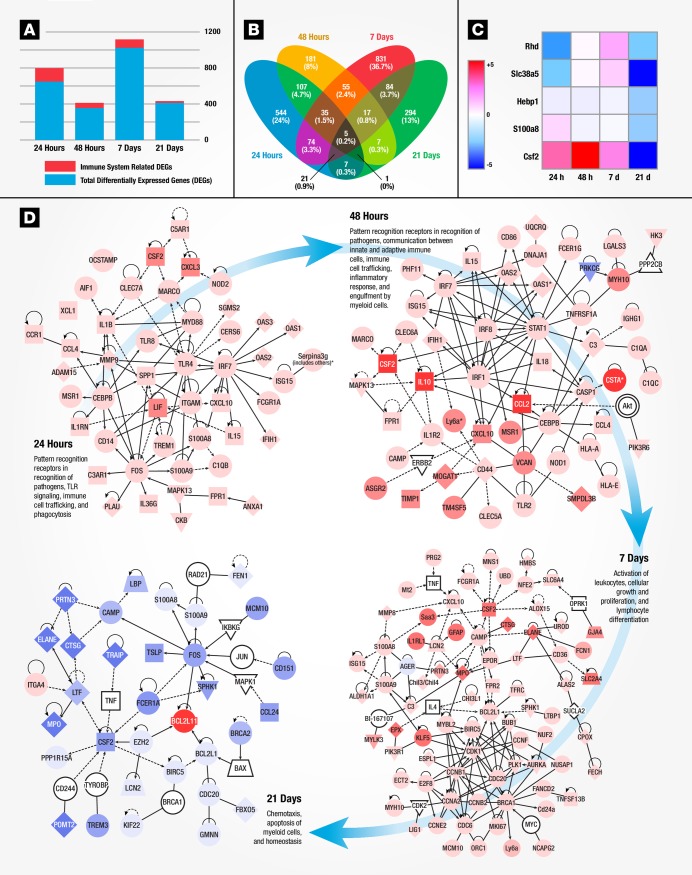
Early gene signatures of immune response to recombinant Sm-p80 + ODN vaccination in mice. **(A)** Differences in DEGs and immune system related mapped genes (y axis) across time-points (x axis) for mouse splenocytes. **(B)** Venn diagram depicting the number and percentage of significant differentially expressed genes (*P* <0.01) at 24 hours (blue), 48 hours (yellow), 7 days (red), or 21 days (green) post-vaccination are shown. Genes that are common to multiple time points are shown by the overlap. **(C)** Heat map analysis for 5 differentially expressed splenocyte genes (rows) common to all time points examined from 24 hours to 21 days (columns) post-rSm-p80 immunization. **(D)** Immune system related networks derived from RNA transcripts at each time-point post-rSm-p80 + ODN immunization. Differentially expressed genes identified at each time point were imported into IPA, and the list of genes identified from top regulator effects were selected for molecular network interactions. The overall interaction of each network is described as different biological processes at each time point. Differences in expression values are represented as red for higher fold change or blue lower fold change.

It is clear that vaccines induce robust adaptive immunity by triggering a vigorous innate immune response that directs the type of induced protection (cell mediated or humoral) [24, 25]. We therefore investigated the early time points during the developing adaptive immune response to identify key gene signatures of rSm-p80 + ODN vaccination. Mice were vaccinated once and splenocytes were analyzed by RNA-Seq at 24 hours, 48 hours, 7 days, and 21 days post-vaccination (Fig 1B) for differences in gene expression (S3 Dataset, S2 Fig and S3A Fig). The highest number of immune system related DEGs was observed 24 hours post-vaccination (Fig 3A). At 24 and 48 hours, we found a vigorous innate immune response including the induction of pattern recognition genes (S100A family genes, *TLR4*, *8*, *13*, and NOD-like receptors), inflammatory cytokines (CSF family, IL-1 family, *IL-15*, *IL-18*, and TNF superfamily), increased expression of *IRF*s *1* and *7* with induction of several interferon inducible/regulated genes, and increased expression of complement factors and antigen presenting genes (S3B Fig). We found an increase expression of *IL-27*, a cytokine related to IL-12A, which promotes the expansion of naïve CD4+ T cells and drives TH1 differentiation [26]. At day 7, most upregulated genes were involved in adaptive immune responses such as immunoglobulin re-arrangement (increased expression of IghG1, 2, 3 and IghE heavy chains) and cell proliferation. By 21 days, there were fewer DEGs with most being down regulated (S2 Fig and S3 Fig). Five genes were found to be linked between 24 hours to 21 days post-vaccination time points and may play a role in inducing the adaptive immune response to rSm-p80 + ODN vaccination (Fig 3B and 3C). Altered Colony Stimulating Factor 2, *CSF2*, expression was detected starting at 24 hours to 21 days with peak expression at 48 hours (8.5-fold increase). These 5 genes, *CSF2*, *S100A8*, *Hebp1*, *Slc38a5*, and *RhD* are involved in complex molecular networks of many immune system related genes, pointing to a possible role of these genes in initiating adaptive responses to Sm-p80 vaccination (S4 Fig). Epistatic interactions in mice immunized with rSm-p80 + ODN revealed major molecular networks of immune system-related gene expression altered at each time point examined (Fig 3D). At 24 hours, antigen recognition appears to be mediated by a major node of interaction, the Toll-like receptor *TLR4*, and its signal transducer adapter molecule *MYD88* and transcription regulator *FOS* resulting in the expression of pro-inflammatory cytokines and IFN-inducible genes; which in overall mediate early immune response leading to an increased inflammatory responses and communication between innate and adaptive immune cell-related genes observed at 48 hours [27, 28]. At day 7, activation of leukocytes and cellular growth and differentiation processes appeared to be mediated by the cell cycle regulatory molecules *CCNB1*, *CDK1*, *CCNA2*, *BRCA1*, and *CDC20*. Homeostasis and contraction of immune system processes were observed at day 21. The observed homeostasis was induced by downregulation of *FOS*, and upregulation of *BCL2L1*, which acts as both an anti- and a pro-apoptotic regulator [29]. Altogether these networks display the classical phases of an adaptive immune response with: antigen recognition and presentation, lymphocyte activation and differentiation, and homeostasis (S5 Fig).

### Gene signatures of immune responses to vaccination with rSm-p80 + ODN compared to wild type *Schistosoma mansoni* infection in mice

**Fig 4:**
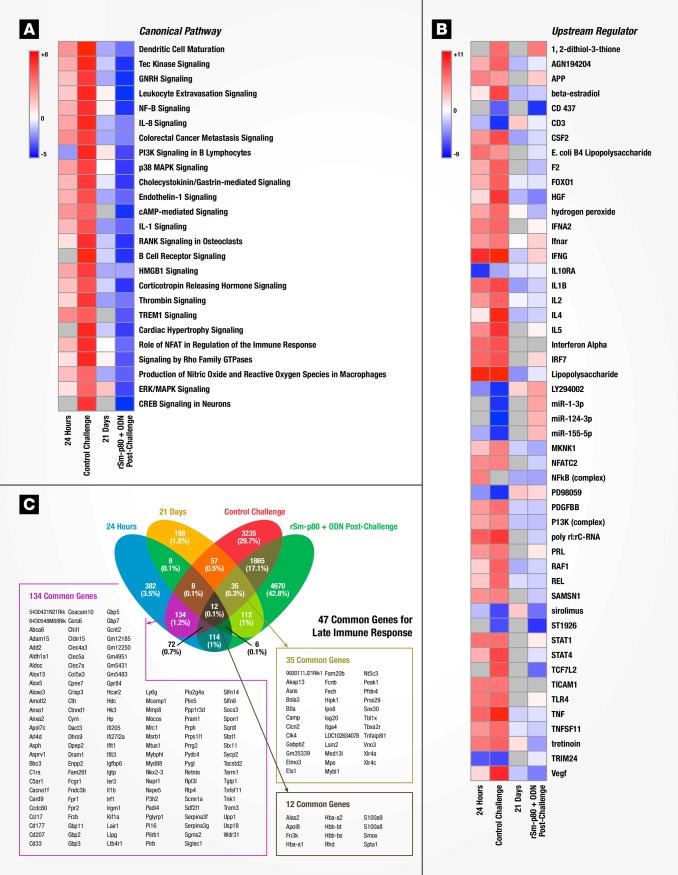
Molecular signatures of immune responses to vaccination with rSm-p80 + ODN compared to wild type *Schistosoma mansoni* infection. **(A)** Top 25 identified canonical pathways. Predicted activation status (z-score) for a given pathway is represented as red (activation) or blue (inhibition). **(B)** Top 50 identified upstream regulators with predicted z-score of activation. **(C)** Venn diagram of shared genes across datasets.

**Fig 5:**
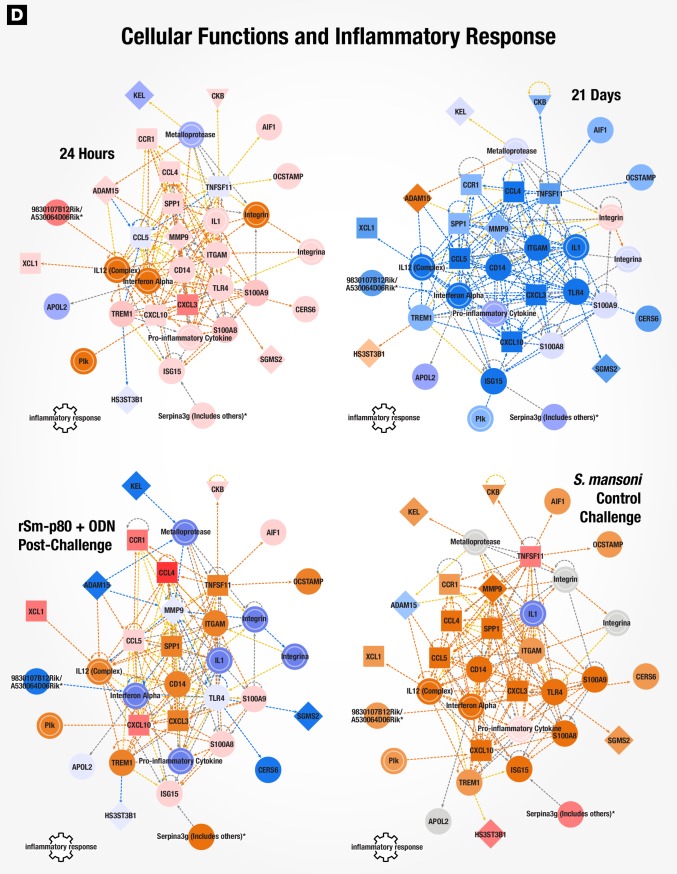
Cellular functions and inflammatory response network. IPA identified cellular functions and inflammatory response network at 24 hours post-vaccination. Expression values for 21 days, rSm-p80 + ODN post-challenge, and *S*. *mansoni* control challenge datasets were overlaid with predicted activation scores.

**Fig 6:**
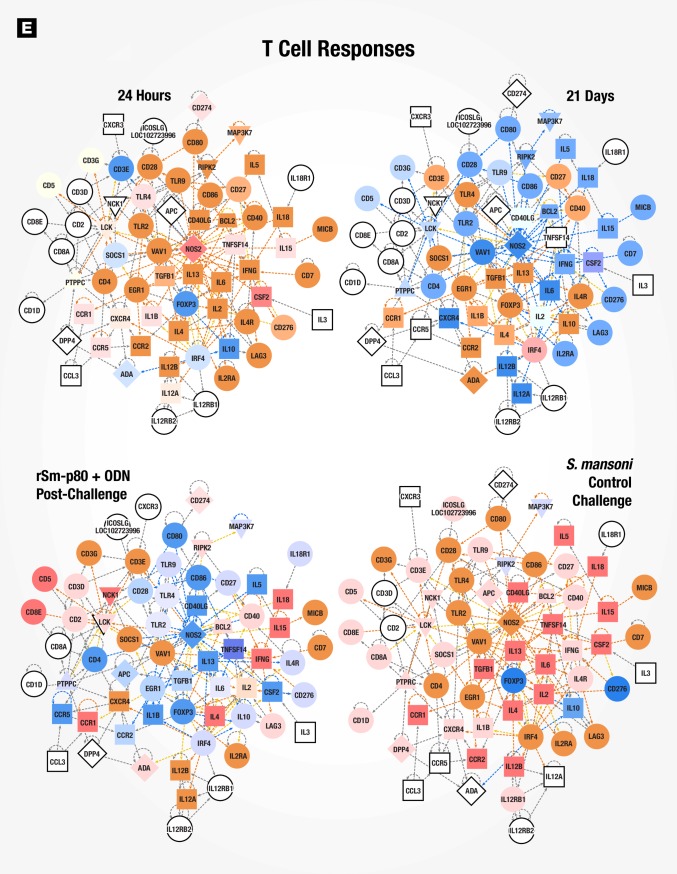
Classical T cell response gene network. Classical T cell response related gene network with overlaid expression values and activation scores.

**Fig 7:**
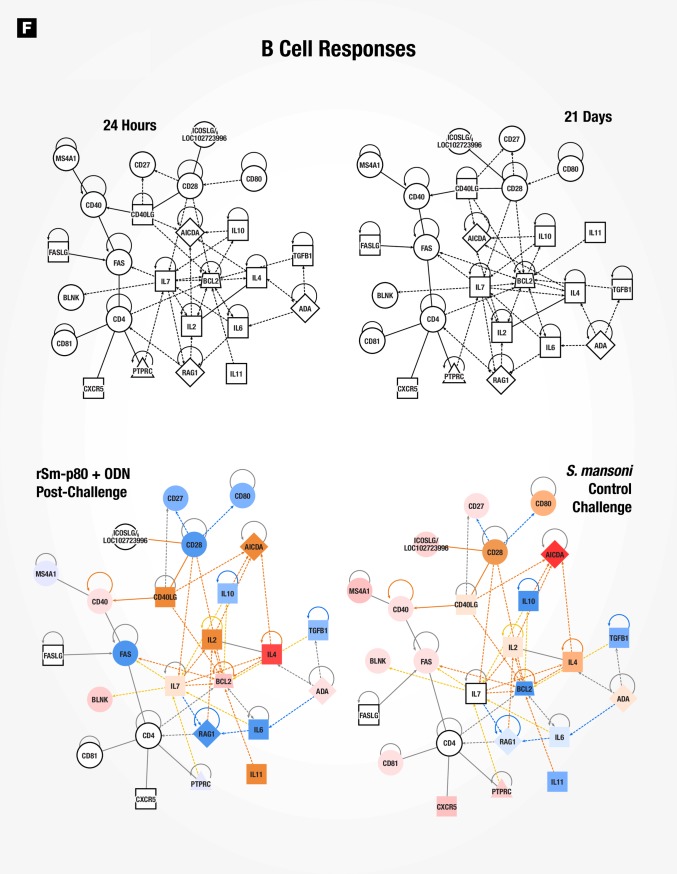
Classical B cell response gene network. Classical B cell response related gene network with overlaid expression values and z-score of activation.

Surface antigenic remodeling is a mechanism employed by helminths to evade the host immune system [8, 30]. Since Sm-p80 is important in membrane biogenesis of the parasite, we compared datasets for Sm-p80 vaccination alone (24 hours and 21 days) to *S*. *mansoni* infection in naïve mice to determine using gene signatures how closely vaccination mimicked *S*. *mansoni* infection. 24 hours after recombinant Sm-p80 + ODN vaccination, a pattern of altered canonical pathways similar to *S*. *mansoni* infection in naïve mice was seen with activation markers for dendritic cell maturation, Tec kinase signaling, GNRH signaling, and leukocyte extravasation signaling, amongst others (Fig 4A). The activation state of upstream regulators revealed similar patterns in early-induced genes (24-hour post vaccination) compared to control challenged mice (chronic infection), likewise the 21 days post immunized mice were similar to vaccinated then *S*. *mansoni* challenged animals (Fig 4B and 4C). Ingenuity Pathway Analysis (IPA) identified cellular function and inflammatory response processes as one of the top 5 networks; an overlay of corresponding datasets demonstrated similar activation patterns between rSm-p80 + ODN vaccinated mice (24 hours) and *S*. *mansoni* control challenge (Fig 5). Network mapping interaction of well-established T and B cell response related genes [31] demonstrated a high activation of T cell response-related genes at 24 hours post vaccination as well as in control infection, while a more mixed activation/inhibition profile was observed in rSm-p80 + ODN immunized animals at 21 days and after *S*. *mansoni* challenge infection (Fig 6). The presence of the parasite seems to induce more B cell response-related genes since 24 hours or 21 days post-immunization few genes participate in the activation or inhibition of classical B cell responses. Whereas mice infected with *S*. *mansoni* induced the expression of *IL4*, *IL7*, *CD40*, *BLNK*, and *BCL2* in both rSm-p80 + ODN immunized then challenged animals and naïve infected mice (Fig 7).

### Gene signatures identified rSm-p80 + ODN immunized mice in an independent blinded study

**Fig 8:**
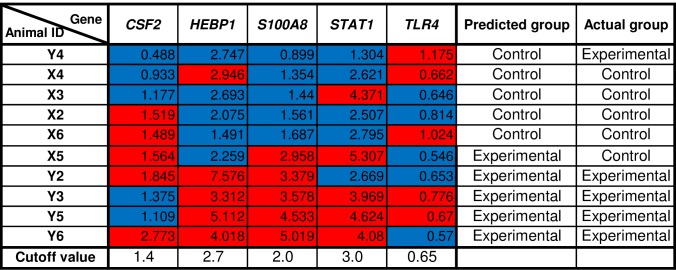
Gene signatures identified rSm-p80 + ODN immunized mice in an independent blinded study. Ten mice were blindly immunized with either rSm-p80 + ODN or ODN alone. One-day post-immunization spleens were dissected and RNA isolated to prepare cDNA. Quantitative RT-PCR expression levels of selected genes identified with 80% accuracy the immunization given for each mouse. Relative fold change of *CSF2*, *HEBP1*, *S100A8*, *STAT1*, and *TLR4* for each mouse. Values represent the average of triplicate PCR experiments normalized to corresponding expression value of each gene and *GAPDH* from a naïve mouse. Cutoff value for each gene was assigned based on top 50 percentile for each gene; mice were assigned as control (blue) or experimental (red). The overall gene pattern of the 5 genes was used to predict each mouse as control or experimental; 8 out of 10 mice were correctly identified.

In order to test the gene signatures potential to correlate with Sm-p80 immunization; we blindly immunized ten mice with either rSm-p80 + ODN or ODN alone. Twenty-four hours post-immunization, mice spleens were processed to extract RNA for qRT-PCR analysis of *CSF2*, *HEBP1*, *S100A8*, *STAT1*, and *TLR4*. Based on the top 50% expression value for each gene a cutoff value was assigned to identify each as control or experimental. The overall expression pattern for the 5 genes was used to identify each animals’ treatment. In line with the idea to use the signatures to predict vaccine efficacy, we achieved 80% accuracy in predicting protection in rSm-p80 + ODN immunized mice (Fig 8).

### Epistatic interactions in baboon peripheral blood mononuclear cells and secondary lymphoid tissues after rSm-p80 + ODN vaccination and *S*. *mansoni* challenge

**Fig 9:**
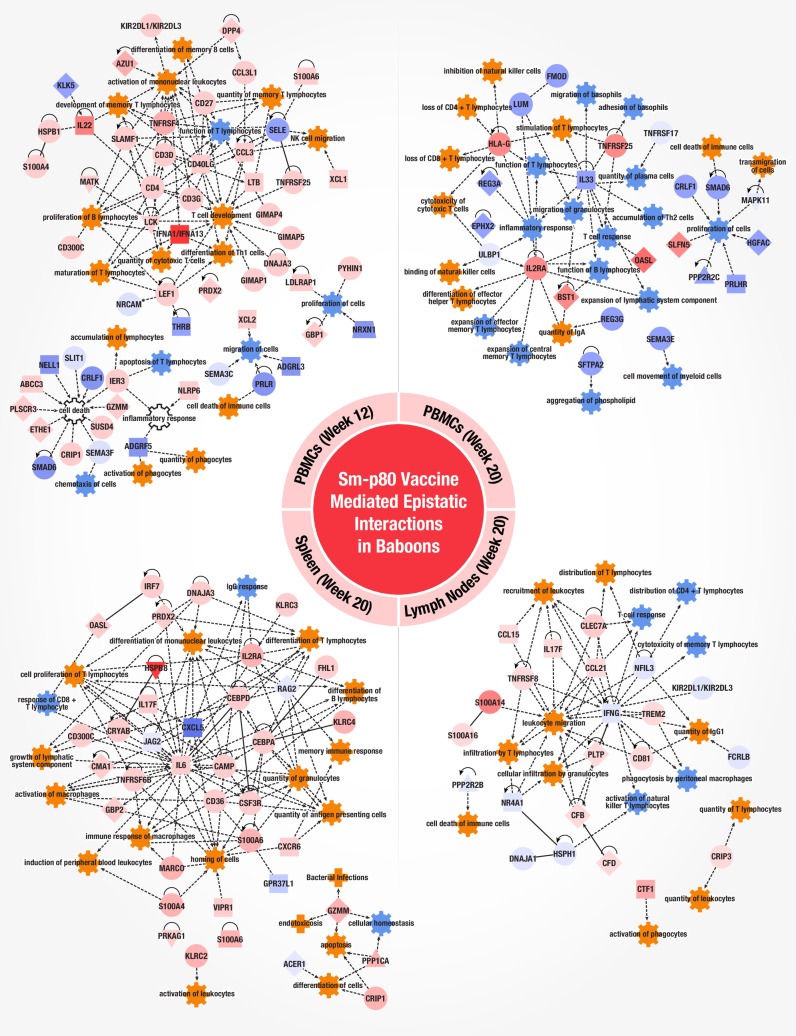
Gene networks in baboon PBMCs and secondary lymphoid tissues after rSm-p80 + ODN vaccination and S. *mansoni* challenge. Gene ontology classification identified genes related to an immune system process for each tissue. Immune system process genes were imported into IPA to connect molecules and identify biological activities triggered by the gene networks. Immune system functions were mapped to identify the overall interactions and predicted activation states (activated = orange, inhibited = blue). Differences in expression values among individual genes are color coded (red = increased, blue = decreased fold change).

In order to develop a safe schistosomiasis vaccine for human use it will be essential to assess vaccine efficacy and identify correlates of protection in a biologically relevant animal model. We selected baboons (*Papio anubis*) in this study since they share greater physiological and immune system resemblance to humans as well as being a natural host for schistosomiasis [32]. In this baboon model of infection, we studied epistatic interaction after rSm-p80 + ODN vaccination. Baboons were immunized three times with rSm-p80 + ODN (n = 4) or ODN alone (n = 4) and peripheral blood mononuclear cells (PBMC) were collected for baseline immune signatures immediately post vaccination as well as just prior to *S*. *mansoni* challenge. Animals were euthanized at week 20—after establishment of the disease—to assess worm burden and collect PBMCs and secondary lymphoid tissues. Worm burden protection was observed to be 58% in vaccinated animals [9]. Tissue mononuclear cells (MNCs) were analyzed by RNA-Seq to identify Sm-p80 vaccine-mediated epistatic interactions (Fig 1C). Statistically significant DEGs varied across tissues with spleen at week 20 displaying the highest number of DEGs (8,637 *p*<0.1 and >2-fold) (S4 Dataset, S6 Fig and S7A Fig). Expression patterns across MNCs in circulation (Week 20 PBMCs) and secondary lymphoid organs were observed with 12 genes in common across all tissues (S7B and S7C Fig). Even in the presence of the parasite, baboon PBMCs at weeks 12 and 20 had almost identical expression values (S7D Fig). Similarly, PBMC and spleen at week 20 demonstrated similar expression patterns for certain common genes (S7E Fig). The most salient difference was observed between the expression values found in PBMC versus mesenteric lymph nodes (S7F Fig); as well as spleen and lymph node differences in gene expression (S7G Fig) Pathway analysis identified 310 significant canonical pathways to overlap with genes from the baboon tissue data sets (S2 Table). Most pathways were found to be unique for each tissue except for agranulocyte and granulocyte adhesion and diapedesis, crosstalk between DCs and NKCs, EIF2 signaling, HMGB1 signaling, and the production of nitric oxide and reactive oxygen species in macrophages which were differentially expressed pathways in common to at least two tissues. To further identify gene predictors of vaccine immunogenicity, immune system process related genes were mapped into networks for each tissue (Fig 9). PBMC (week 12) gene networks identified included the activation of T cell development, differentiation of TH1 cells, maturation and memory of T lymphocytes by nodes of interaction for the genes *CD40LG*, *LCK*, *LEF1*, *IFNAA1/A13*, *IL-22*, *TNFSRSF4*, *LTB*, *CCL3* and *CD27*. A distinct phenotype was observed in PBMC (week 20) after infection with *S*. *mansoni—*most likely due to the immune recall response and clearance of the pathogen. Although there was still differentiation of effector helper T cells and transmigration of cells; downregulation of major nodes *IL33*, and upregulation of *IL2RA*, *HLA-G*, and *TNFSRSF25* orchestrated a putative inhibition of inflammatory responses, cell proliferation, inhibition of expansion of effector and central memory T lymphocytes differentiation, loss of lymphocytes, and cell death of immune cells. Tissue specificity was observed in secondary lymphoid organs, however, we found similar immune system processes of innate and adaptive cellular immunity in PBMC after vaccination (week 12). Downregulation of the *IFNG* was observed to play a role in lymph nodes with *IL-17F*, *CCL21*, *TNFRSF8*, *PLTP* and *CLEC7A* mediating the predicted activation of recruitment, distribution, and migration of leukocytes; *IL-17F* has been shown to stimulate pro-inflammatory cytokines *IL-6*, *IL-8* and *GM-CSF2* [33, 34]. The spleen transcriptome displayed altered gene networks as well with *IL6* as a major player interacting with *CAMP*, *CEBPA*, *CSF3R*, and *TNFRSF6B* to activate the expansion of lymphatic systems, induction of PBMC, cell homing, and differentiation of T and B lymphocytes. Altogether, the transcriptomes of Sm-p80 immunized baboon tissues displayed activation and proliferation of both innate inflammatory elements (*S100A* family, *IRF7*, *IL-17*, *IL-6*, *INFA*) and adaptive cellular TH1 helper cells, CD8 T cells, and humoral responses with B cell differentiation. Both PBMC at 12 weeks post vaccination and spleen at 20 weeks showed predicted gene signatures for memory immune responses.

## Discussion

rSm-p80 + ODN-based vaccines demonstrated the highest levels of protection as well as higher number of DEGs compared to the other Sm-p80 formulations. Comparison of the common 59 genes shared across all vaccine formulations and *S*. *mansoni* challenged naïve mice revealed up-regulation of nearly all genes in the control challenged group while most genes were down-regulated for vaccinated animals. Thus pointing to a similar transcriptome response induced by the vaccine and natural infection, with vaccination leading to lessened expression of *S*. *mansoni*-induced genes at time of infection.

Likewise, rSm-p80 + ODN immunization elicited a more robust immunological response at the transcriptional level compared to other Sm-p80 formulations. Surprisingly, in the presence of ODN, a TLR9 agonist, early-gene responses to Sm-p80 + ODN demonstrated upregulation and gene interactions that are associated with the innate lipopolysaccharide (LPS) sensor *TLR4*. We found that both vaccination and control infection induced upstream regulators associated with recognition of LPS [35]. If TLR4 is central to protective immune responses to *Schistosoma spp*., adjuvant formulations including a TLR4 agonist may be preferred for vaccines against these diseases. This is consistent with our findings of durable protection against parasite challenge with the SchistoShield^®^ vaccine, a TLR4-based adjuvant formulation, GLA-SE [36, 37], combined with rSm-p80 [10].

The presence of *CSF2* appears to mediate an important role during the initial response to Sm-p80. GM-CSF, the protein product of *CSF2*, is involved in a wide range of biological processes in both innate and adaptive immunity, with its production being tightly linked to the response to danger signals and hematopoiesis. It has also been demonstrated that Dendritic Cells (DCs) generated in the presence of GM-CSF plus IL-15 prime potent CD8+ TH1 responses [38] and GM-CSF has been used as an adjuvant to enhance B and T helper cell activity with vaccines [39, 40]. Signaling molecule, *S100A8*, and heme-binding protein 1, *Hebp1*—both molecules involved in calcium signaling in immune cells—were up-regulated at 24 hours and are known to play a role in danger signaling and chemotaxis of monocytes and DCs [41, 42]. Sodium coupled neutral amino acid transporter 5, *Slc38a5*; and blood group Rhesus factor, *RhD* were found to be increased only at day 7. The roles of *Slc38a5* and *RhD* are not as clear and may be limited to normal cellular development during expansion of the adaptive immune response.

The identification of *CSF2*, *HEBP1*, *S100A8*, *STAT1*, and *TLR4* as potential vaccine predictors support our data analysis in the identification of key genes to be associated with Sm-p80 vaccination. These results should be further evaluated in future schistosomiasis vaccination studies as well as during Sm-p80 vaccine clinical trials.

Common gene(s)/activation pathways were found to be present in data sets of both mice and baboon splenocytes. These can be summarized as detection (alarmins), migration (colony stimulating factors), and response (tumor necrosis factor receptors). The macrophage migration *CSF* genes appears to play a key role as may be expected for primary mediators of phagocytic cell guidance signals. Additionally, S100A alarmin genes are a common signature of vaccine efficacy as *S100A8/9* in mice and *S100A4/6* in baboon are found to participate in key gene networks. Finally, *TNFRSF* genes appear to also play a role in mediating vaccine efficacy as *TNFRSF6B* in baboon spleen, *TNFRSF25* in baboon PBMCs, and *TNFS1A/B* in mice were observed to be involved in network interactions. The identification of *TNFRSF*-related genes in mediating vaccine efficacy has been previously reported, as the *TNFRF17* gene is a predictor of antibody responses to the YF-17D and TIV [11, 13]. Contrary to the results observed with infection by normal or radiation-attenuated cercariae; the predominant early immune response was TH1 mediated and aimed at the adult worm; then after egg deposition TH1 responses diminished and were replaced by TH2 mediated response [43]. Here, we found that rSm-p80 + ODN vaccination induced gene networks associated with both cellular and humoral responses—predominantly TH1 biased–with some TH2 and TH17 gene signatures in both mice and baboons.

Systems biology approaches using data integration from different animal models immunized with Sm-p80 permitted the observation of a global transcriptome profile of vaccine-induced immune responses and the identification of epistatic interactions following *S*. *mansoni* challenge. RNA-Seq comparisons provided a method for inferring key genes involved in mediating rSm-p80 vaccine immunogenicity. As bioinformatics tools have become more accessible, this methodological approach can now be applied to investigate–and possibly predict—immunogenicity for novel non-licensed vaccine candidates prior to and during human clinical trials enabling more rapid decisions on further human development of new vaccine candidates.

## Supporting information

S1 Checklist (PDF)

S1 Dataset Raw reads sequencing data were deposited in 4 different BioProjects at NCBI; each BioProject encompasses all different samples used in each sequencing run with file names summarized. (XLSX)

S2 Dataset List of statistical significant differentially expressed genes from splenocytes of mice immunized with Sm-p80 vaccine formulations and challenged with *S*. *mansoni*. Each sheet contains the list of DEGs for each experimental group compared to its corresponding control group. Each table contains Gene ID, linear fold change, Log base 2-fold change, RPKM values for each group, standard deviation, P-value, and T-value. Differentially expressed genes identified where *P*<0.01 and greater than 2-fold change.
(XLSX)

S3 Dataset List of statistically significant differentially expressed genes (*P*<0.01 and greater than 2-fold change) from splenocytes of rSm-p80 + ODN immunized mice at different time points post-vaccination (24 hours, 48 hours, 7 days, and 21 days). (XLSX)

S4 Dataset List of statistically significant differently expressed genes (P<0.1 and greater that 2-fold change) in rSm-p80 + ODN immunized baboon tissues (PBMCs, spleen, and lymph nodes). (XLSX)

S5 Dataset Per base quality scores for all sequencing rung. Quality scores across all bases were analyzed using FastQC for all RNA-sequencing runs performed. A representative sample from each run reflects the overall quality score for each of the sequencing runs: (A) Formulations experiment, (B) time-points experiment, (C) baboon peripheral blood mononuclear cells at 12 weeks, (D) baboon peripheral blood mononuclear cells at 20 weeks, (E) baboon lymph nodes, and (F) baboon spleens.
(PDF)

S6 Dataset List of primer nucleotide sequences used for mouse spleen qRT-PCR. (PDF)

S1 Fig Heat map of splenocyte DEGs of transcriptome comparison for Sm-p80 vaccine formulations and challenge. **(A)** Heat map for common immune related genes. **(B)** Heat map for metabolic and cellular process genes correlated to adaptive immune responses. The heat map colors represent the average expression of mice (n = 10 per group) for each vaccine formulation.
(PDF)

S2 Fig Statistically significant differentially expressed genes during the developing immune response post rSm-p80 + ODN immunization in mice. CIRCOS plot for each time-point examined (*p*<0.01 and >2-fold change) during the developing immune response, the colored bar on the outermost layer represents the total number of DEGs with gene names. Blue indicates 24 hours, yellow 48 hours, red 7 days, and green 21 days. Inner layer depicts fold change of each corresponding gene, where red is up-regulation and blue is down-regulation. Inter-connecting links represent DE transcripts that are shared between time points examined; where green indicates overlaps between 24 hours and 48 hours; orange for 48 hour and 7 days; yellow for 7 days and 21 days; aqua for 21 days and 24 hours; violet for 24 hours and 7 days; and light-green for 48 hours and 21 days.
(PDF)

S3 Fig Molecular signatures induced by rSm-p80 + ODN vaccination and *S*. *mansoni* challenge in mice. **(A)** Heat map showing kinetics of changes in expression of genes (rows) across time points (columns). Genes were hierarchically clustered (one minus Pearson correlation) using GENE-E analysis software. Gene clusters of interest are enlarged. **(B)** Gene signatures of innate and adaptive immune responses. Genes were grouped by families and/or functions. The data shows a robust innate immune response (24 and 48 hours) that drives an increase in metabolic and adaptive immune responses (7 and 21 days). The heat map colors represent the average expression of mice (n = 5 per group) for each time point.
(PDF)

S4 Fig Gene interactome for 5 identified common DEGs. The network shows the dynamic interactions of the 5 genes (square nodes) common to all time points with known genes (round nodes). Genes nodes that were identified to be up/down regulated over the 21 days after vaccination are shown in red. The genes that are known direct mediators between the 5 genes of interest are shown as diamond nodes. Non-directional Interactions are shown as edges (green lines-controls expression and blue lines-controls state change). Gene pathways were identified using Pathway Commons Network Visualizer and mapped using Cytoscape.
(PDF)

S5 Fig Bioprofiler filtration of genes related to immune system function. At 24 hours we identified a total of 22 genes to interact in activation of antigen presenting cells. Of interest was *CSF2* participating in the activation of more pathways compared to other genes. At 48 hours we observed a predicted macrophage activation as well as activation of T cells with *CSF2* as a major node of interaction. At seven days after immunization, lymphocyte differentiation activities were observed. By day 21, most networking genes were found to be down-regulated. These interactions lead to a *BCL2L1*, *BCL2L11*, *CSF2*, and *P2RX7* mediated homeostasis.
(PDF)

S6 Fig Differentially expressed genes for PBMCs and secondary lymphoid tissues in rSm-p80 + ODN immunized and challenged baboons. Circular visualization, CIRCOS, plot of statistically significant (*p*<0.1 and >2-fold change) differentially expressed genes from vaccinated baboons’ PBMCs at week 12 (red), PBMCs at week 20 (burgundy), spleen (purple), and lymph nodes (blue). Inner layer depicts fold change of each corresponding gene, where red is upregulation and blue is downregulation. Links indicate the transcripts that are shared between tissues.
(PDF)

S7 Fig Transcriptomics comparison of rSm-p80 + ODN immunized and challenged baboons’ spleen, lymph nodes, and peripheral blood mononuclear cells. **(A)** Gene ontology analysis of differentially expressed genes (y axis) according to biological functions (legends). Analysis was performed with PANTHER GO Classification System. Differences in number of genes were observed across baboon tissues (x axis). **(B)** Venn diagrams illustrating genes overlapping across different baboon tissues. Heat maps of genes common across different tissues. Peripheral blood mononuclear cells collected before (week 12) and after (week 20) *S*. *mansoni* challenge infection where compared. Fold change expression values observed across different tissue comparisons: **(C)** PBMC, spleen, and lymph nodes; **(D)** PBMC at weeks 12 and 20; **(E)** PBMC and spleen; **(F)** PBMC and lymph nodes; and **(G)** spleen and lymph nodes.
(PDF)

S8 Fig Reverse transcription quantitative PCR (qRT-PCR) of selected mouse genes. RNA extracted from pooled mouse samples at each time-point were examined in qRT-PCR. Relative fold change of *CSF2*, *CDK1*, *FOS*, *HEBP1*, *S100A8*, *STAT1*, and *TLR4* for ODN and rSm-p80 + ODN groups at **(A)** 24 hours, **(B)** 48 hours, **(C)** 7 days, and **(D)** 21 days post-immunization.
(PDF)

S1 Table Ingenuity Pathway Analysis identified significant canonical pathways across 5 Sm-p80 vaccine formulations and *Schistosoma mansoni* infected naïve mice. Red: *S*. *mansoni* control challenge; Yellow: DNA vaccine (VR1020-Sm-p80); green: prime/boost (pcDNA3 –Sm-p80 + rSm-p80 + ODN); aqua: rSm-p80 + alum; blue: rSm-p80 + GLA; and violet: rSm-p80 + ODN. The symbol “X” indicates identified genes in dataset overlapping with the corresponding canonical pathway.
(PDF)

S2 Table Ingenuity Pathway Analysis identified significant canonical pathways across rSm-p80 + ODN immunized baboon tissues. Lavender: peripheral blood mononuclear cells at week 12 (immunized); purple: peripheral blood mononuclear cells at week 20 (immunized and S. mansoni infected); red: spleen; and blue: lymph nodes. The symbol “X” indicates genes in dataset overlapping with the corresponding canonical pathway.
(PDF)
