# Supplementary figures and images for: Sm-p80-based schistosomiasis vaccine mediated epistatic interactions identified potential immune signatures for vaccine efficacy in mice and baboons
Source: PLoS One. 2017 Feb 13;12(2):e0171677. doi: 10.1371/journal.pone.0171677 (PMC5305113; doi:10.1371/journal.pone.0171677)

# Supplementary Dataset 5

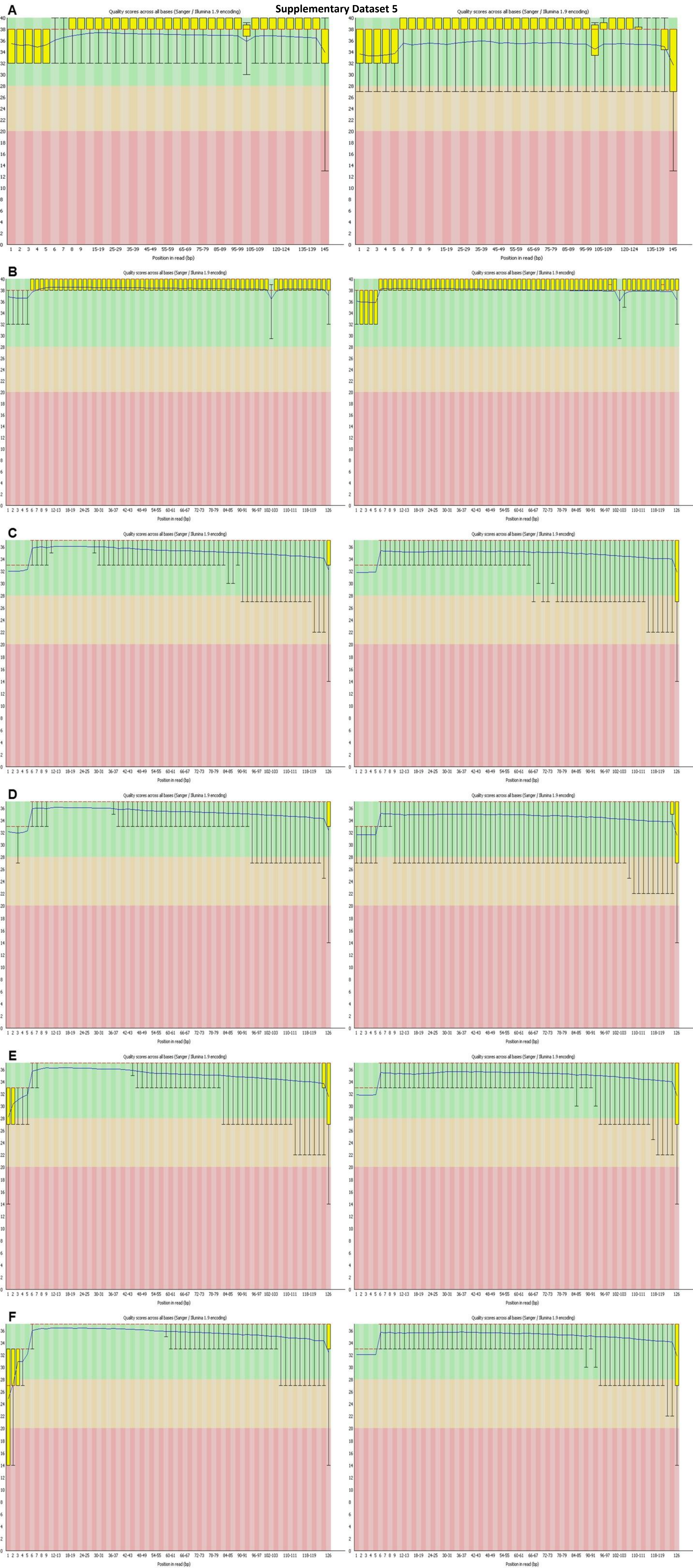

Supplement: S5 Dataset — Quality scores across all bases were analyzed using FastQC for all RNA-sequencing runs performed. A representative sample from each run reflects the overall quality score for each of the sequencing runs: (A) Formulations experiment, (B) time-points experiment, (C) baboon peripheral blood mononuclear cells at 12 weeks, (D) baboon peripheral blood mononuclear cells at 20 weeks, (E) baboon lymph nodes, and (F) baboon spleens. (PDF) [file pone.0171677.s006.pdf]

S1 Fig

A

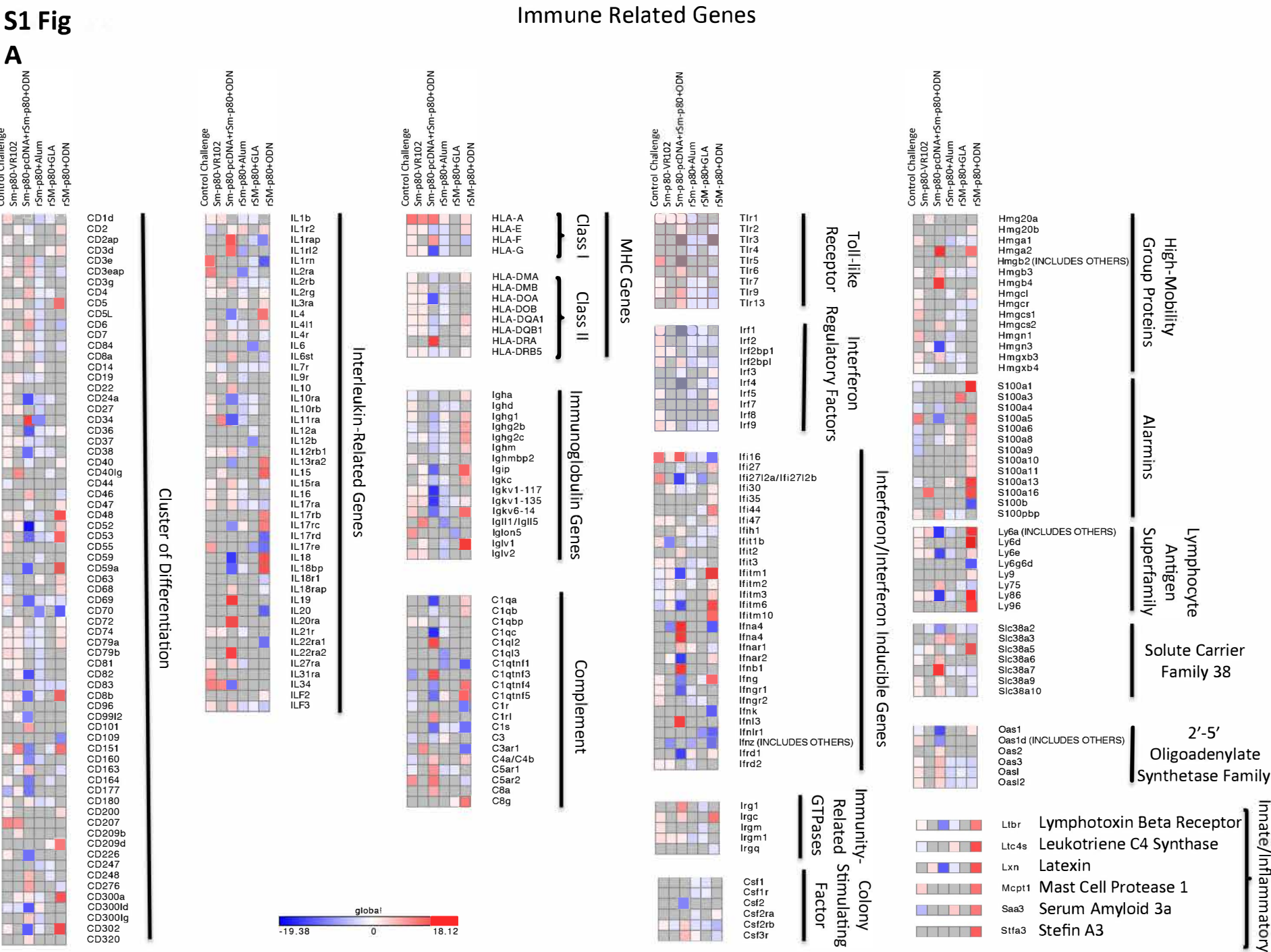

B

## Metabolic and Cellular Process Genes

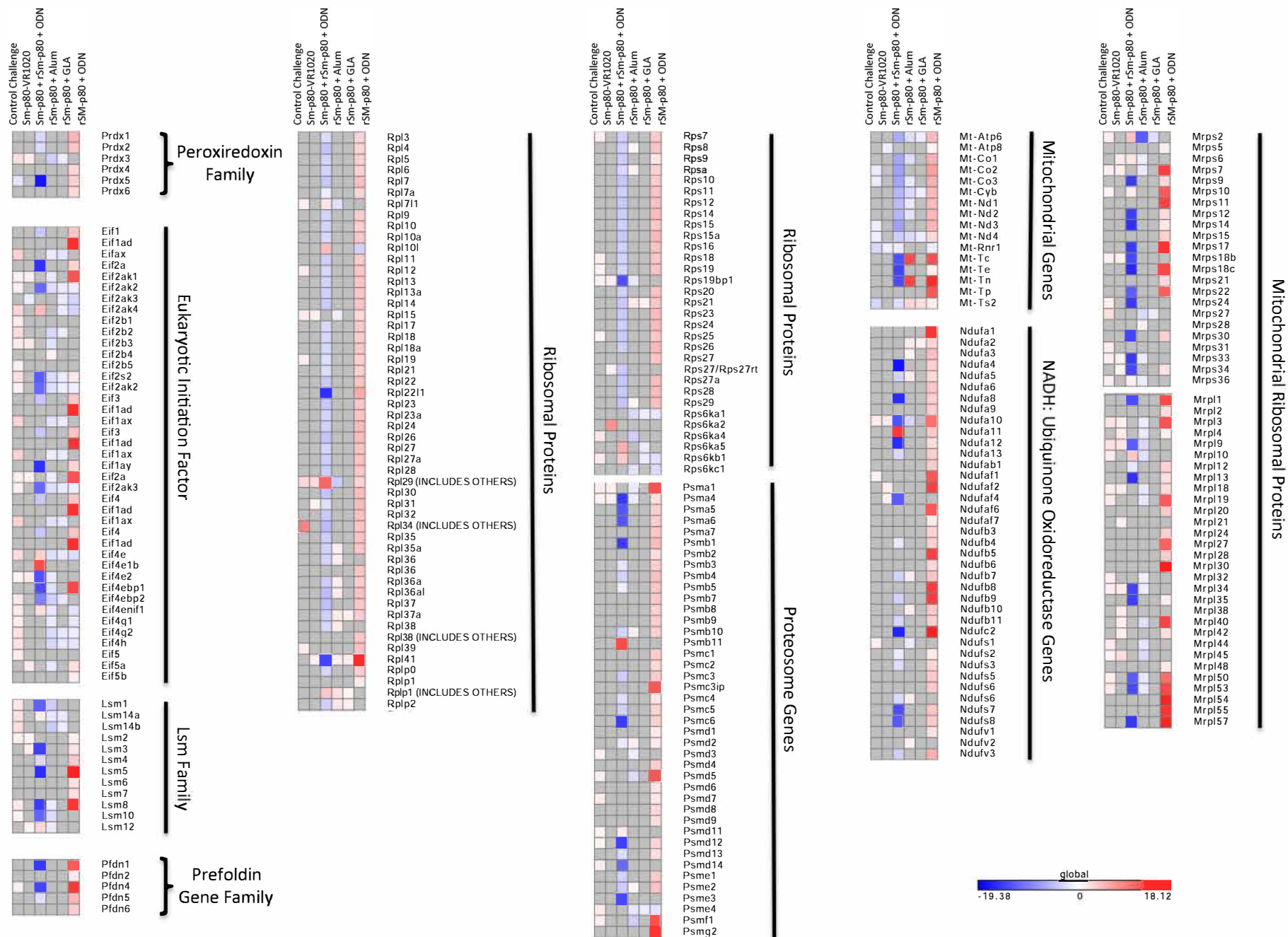

Supplement: S1 Fig — (A) Heat map for common immune related genes. (B) Heat map for metabolic and cellular process genes correlated to adaptive immune responses. The heat map colors represent the average expression of mice (n = 10 per group) for each vaccine formulation. (PDF) [file pone.0171677.s008.pdf]

S3 Fig  
A

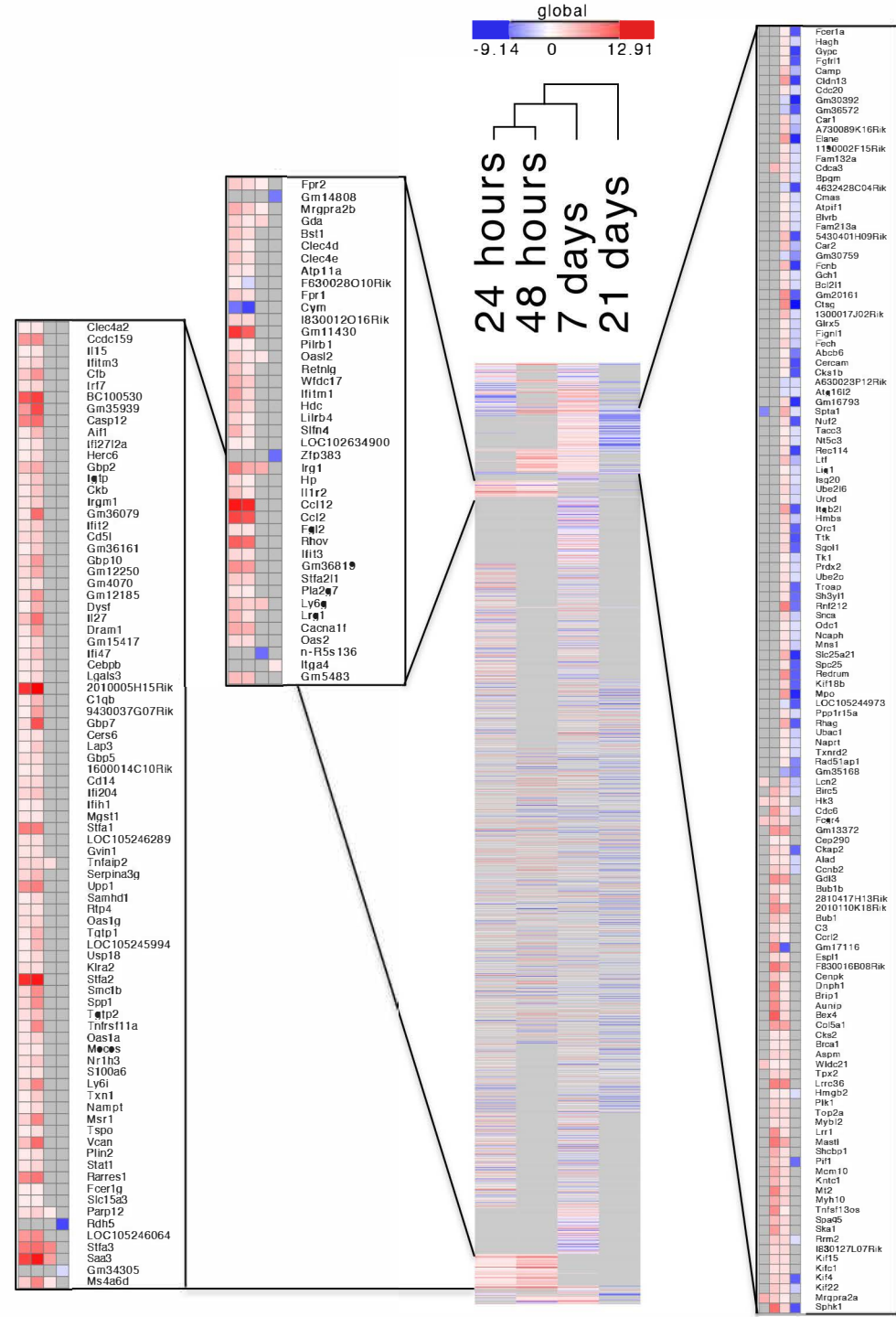

B

Innate Immune Gene Response

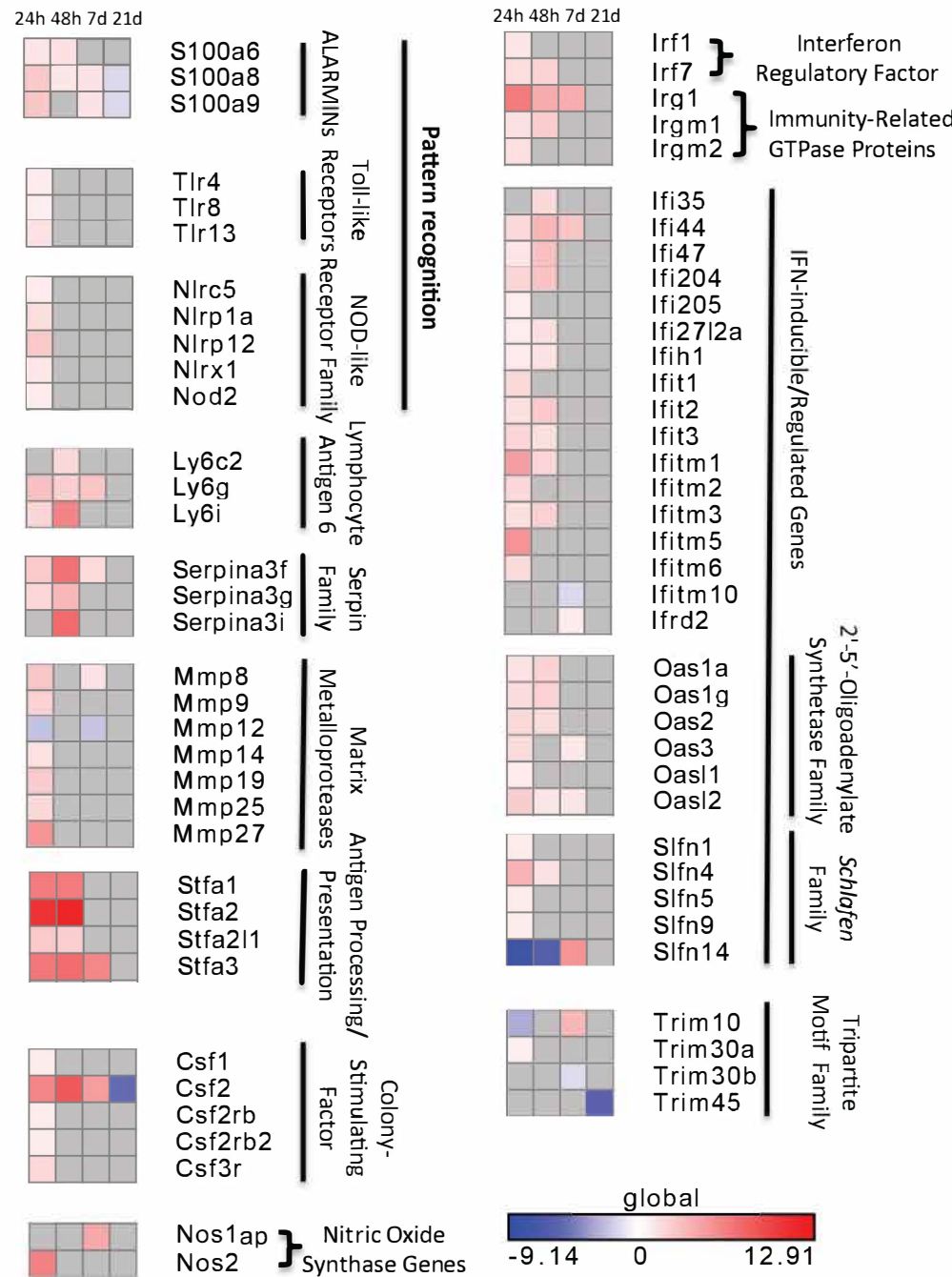

Adaptive Immune Gene Response

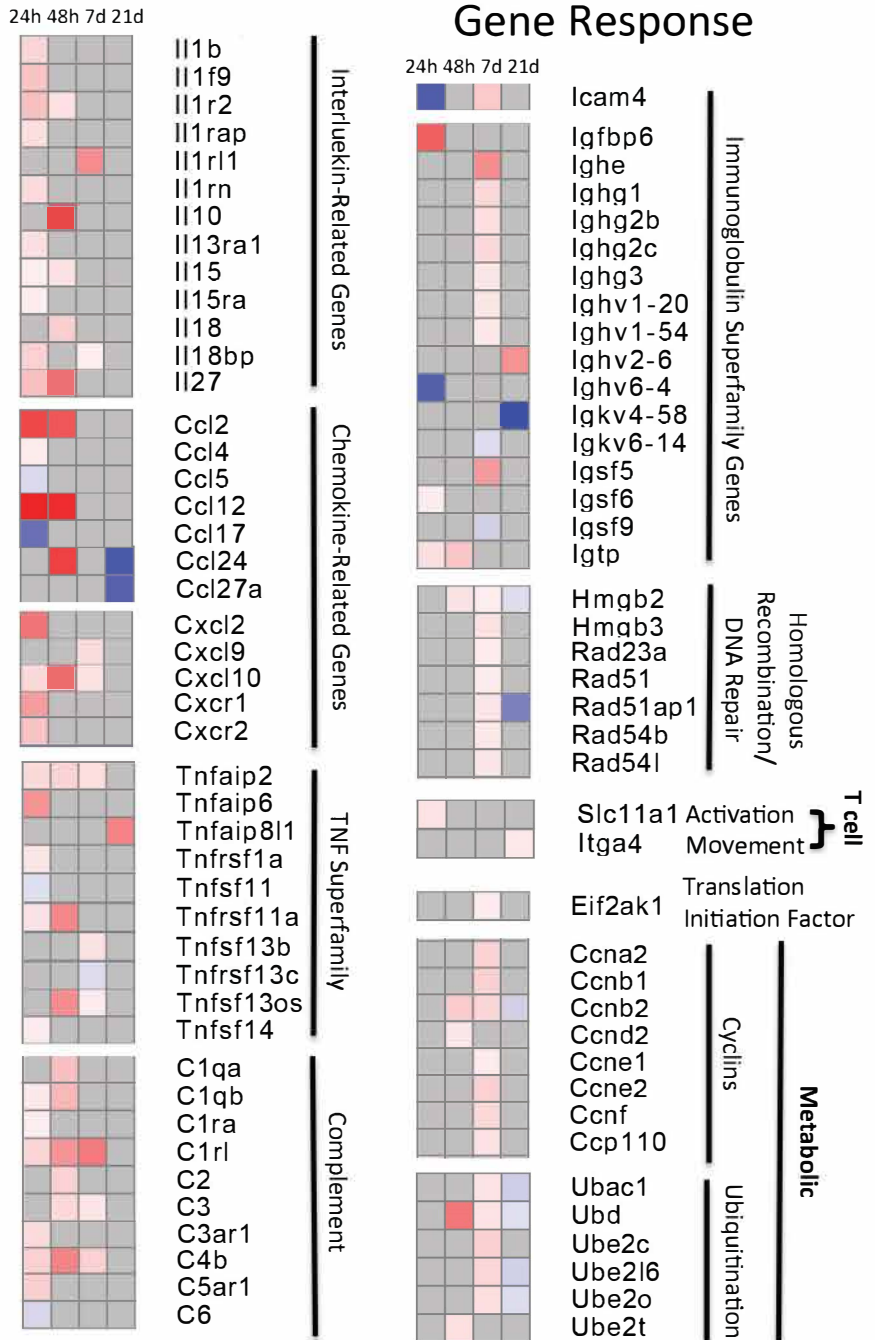

Supplement: S3 Fig — (A) Heat map showing kinetics of changes in expression of genes (rows) across time points (columns). Genes were hierarchically clustered (one minus Pearson correlation) using GENE-E analysis software. Gene clusters of interest are enlarged. (B) Gene signatures of innate and adaptive immune responses. Genes were grouped by families and/or functions. The data shows a robust innate immune response (24 and 48 hours) that drives an increase in metabolic and adaptive immune responses (7 and 21 days). The heat map colors represent the average expression of mice (n = 5 per group) for each time point. (PDF) [file pone.0171677.s010.pdf]

S4 Fig

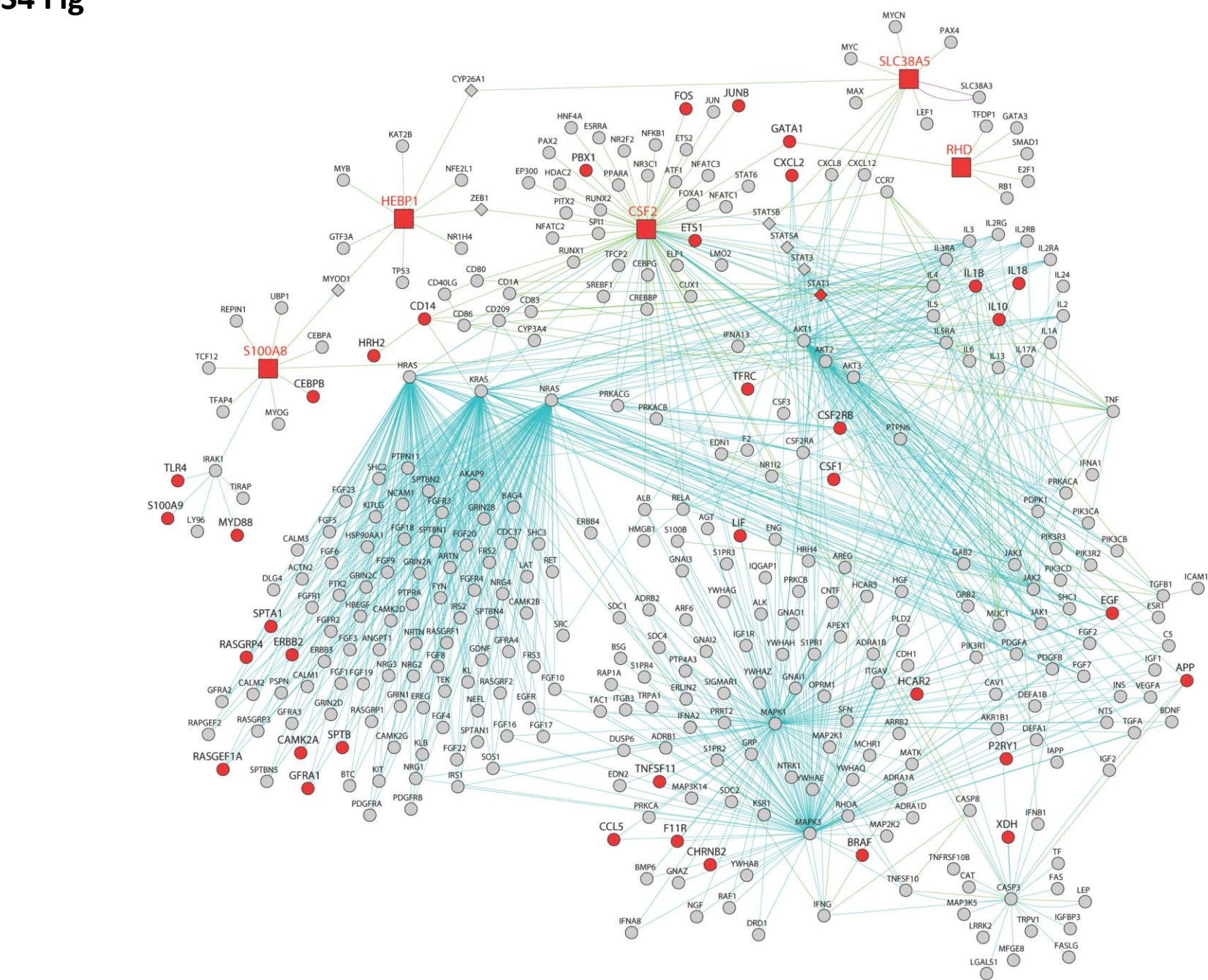

Supplement: S4 Fig — The network shows the dynamic interactions of the 5 genes (square nodes) common to all time points with known genes (round nodes). Genes nodes that were identified to be up/down regulated over the 21 days after vaccination are shown in red. The genes that are known direct mediators between the 5 genes of interest are shown as diamond nodes. Non-directional Interactions are shown as edges (green lines-controls expression and blue lines-controls state change). Gene pathways were identified using Pathway Commons Network Visualizer and mapped using Cytoscape. (PDF) [file pone.0171677.s011.pdf]

## S5 Fig

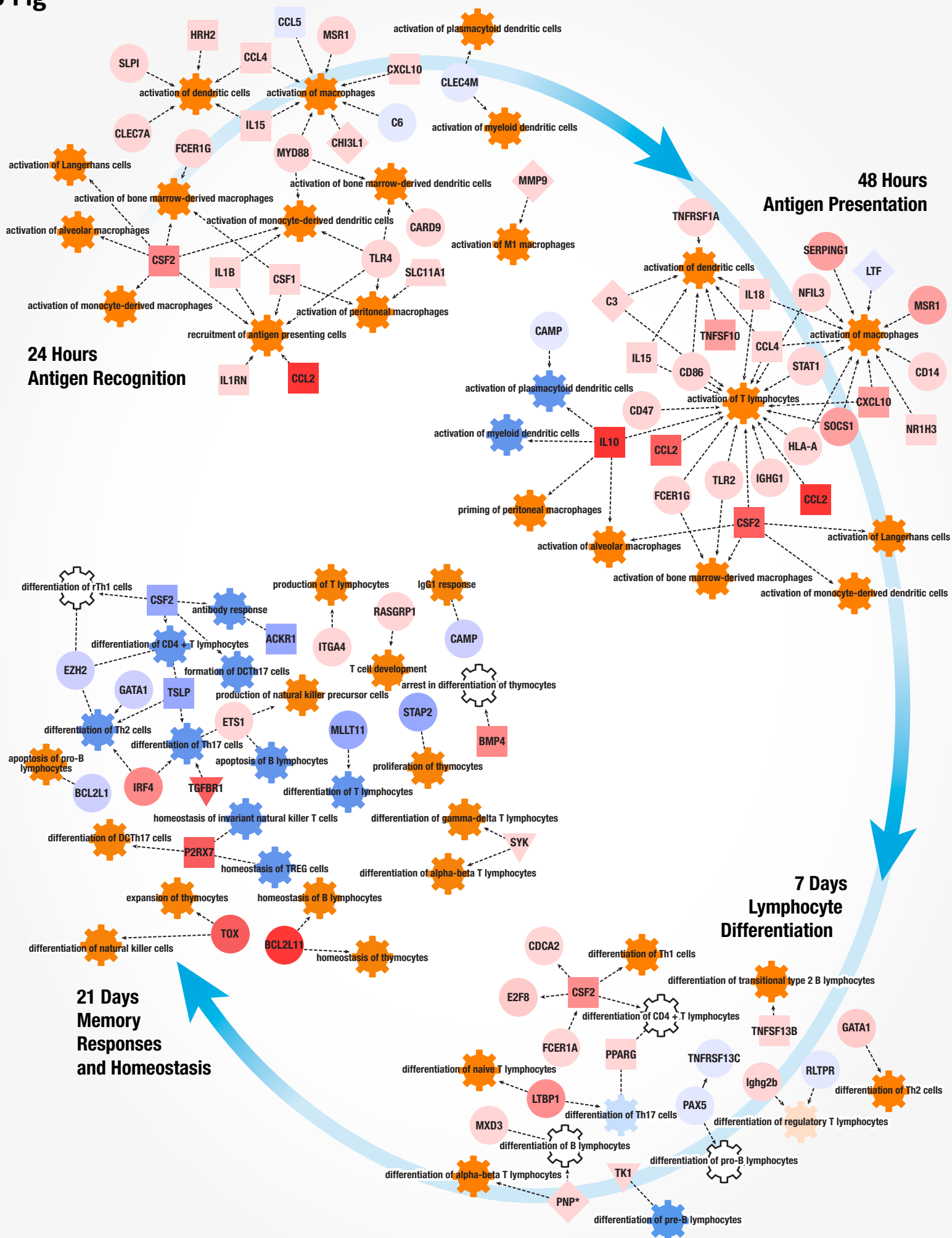

Supplement: S5 Fig — At 24 hours we identified a total of 22 genes to interact in activation of antigen presenting cells. Of interest was CSF2 participating in the activation of more pathways compared to other genes. At 48 hours we observed a predicted macrophage activation as well as activation of T cells with CSF2 as a major node of interaction. At seven days after immunization, lymphocyte differentiation activities were observed. By day 21, most networking genes were found to be down-regulated. These interactions lead to a BCL2L1, BCL2L11, CSF2, and P2RX7 mediated homeostasis. (PDF) [file pone.0171677.s012.pdf]

A

**ODN** **rSm-p80 + ODN**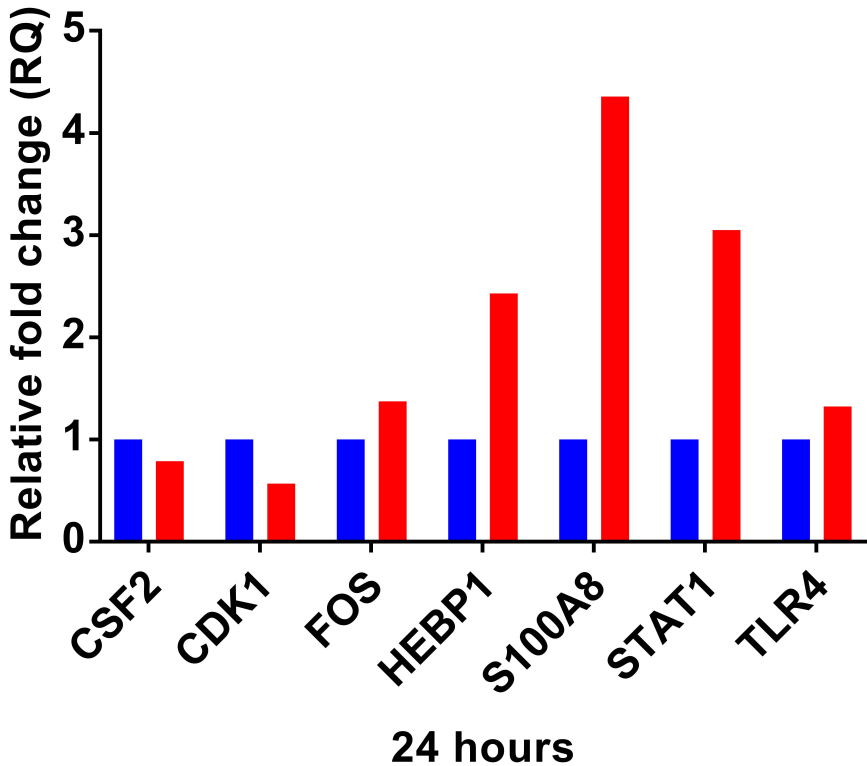

**B**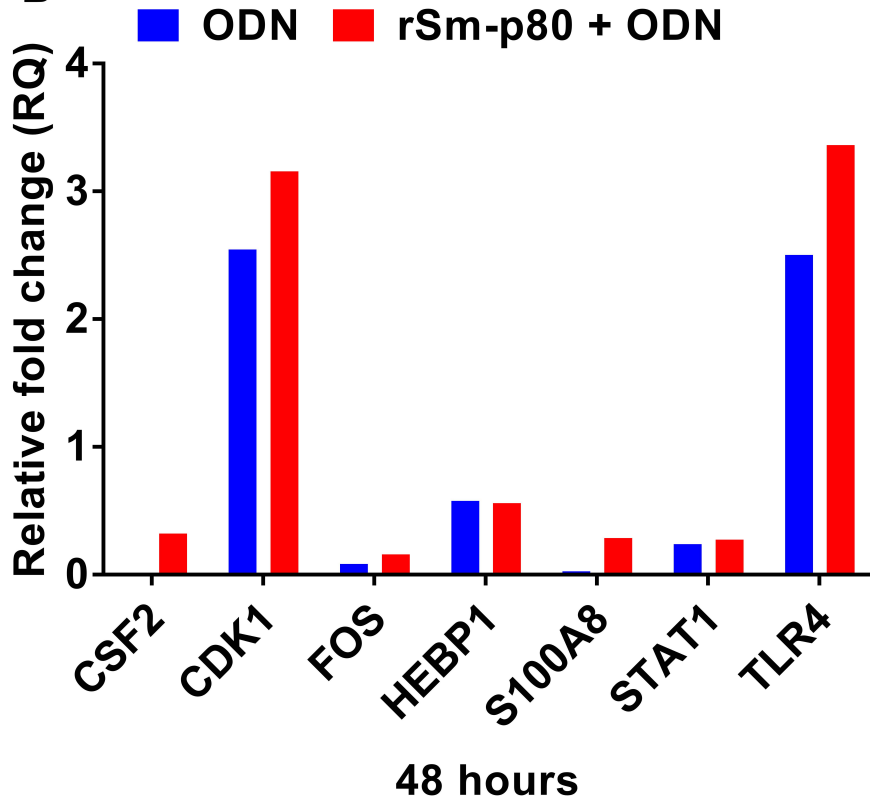

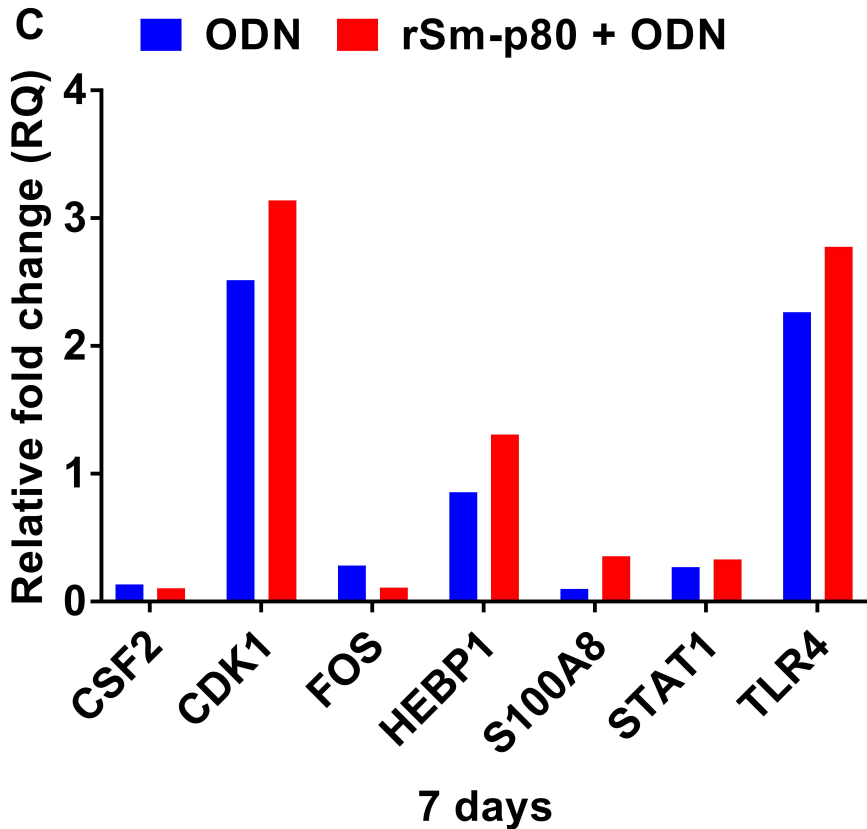

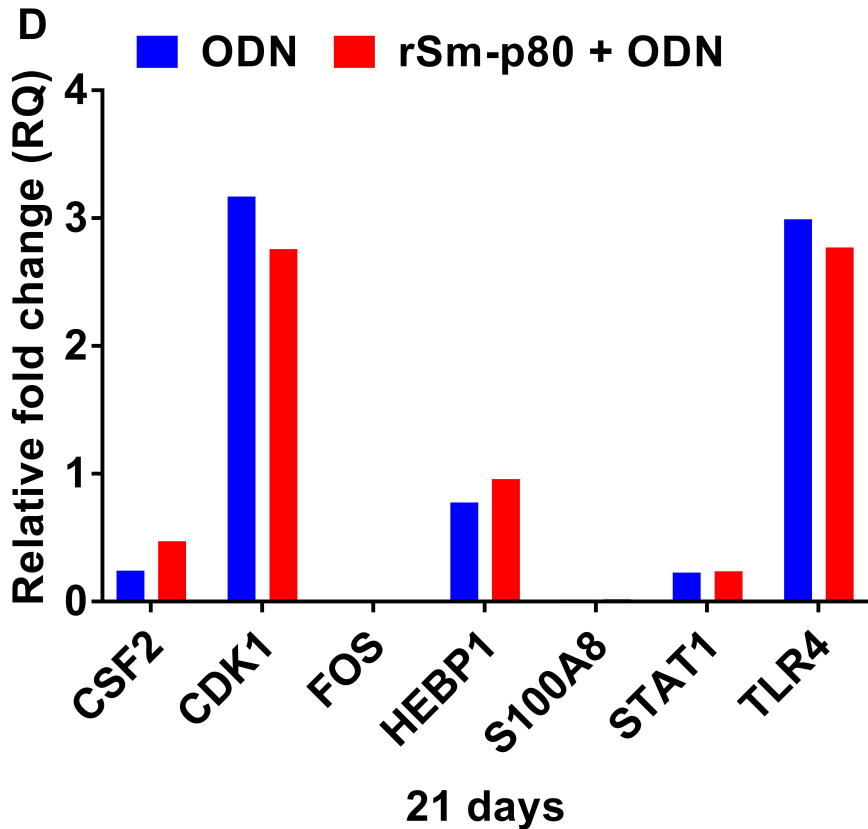

Supplement: S8 Fig — RNA extracted from pooled mouse samples at each time-point were examined in qRT-PCR. Relative fold change of CSF2, CDK1, FOS, HEBP1, S100A8, STAT1, and TLR4 for ODN and rSm-p80 + ODN groups at (A) 24 hours, (B) 48 hours, (C) 7 days, and (D) 21 days post-immunization. (PDF) [file pone.0171677.s015.pdf]
