# Supplementary material for: Sm-p80-based schistosomiasis vaccine mediated epistatic interactions identified potential immune signatures for vaccine efficacy in mice and baboons
Source: PLoS One. 2017 Feb 13;12(2):e0171677. doi: 10.1371/journal.pone.0171677 (PMC5305113; doi:10.1371/journal.pone.0171677)
Supplement: S6 Dataset — (PDF) [file pone.0171677.s007.pdf]

Supplementary Dataset 6

Gene	Primer sequence
CDK1	Forward: 5'-AAG CAG TTG GGA TGA GAA GG-3'<br>Reverse: 5'-CCC TGA TCT CTA GCT GTG AAA C-3'
CSF2	Forward: 5'-AAT GGT GAA GAC CGT GTC AG-3'<br>Reverse: 5'-GGG CAT GGT GAT TTC CTT GTA-3'
FOS	Forward: 5'-GAT GGC TCC CTA CAG AAG AAA C-3'<br>Reverse: 5'-GGA TTC TCC GTT TCT CTT CCT C-3'
GAPDH	Forward: 5'-AAC AGC AAC TCC CAC TCT TC-3'<br>Reverse: 5'-CCT GTT GCT GTA GCC GTA TT-3'
HEBP1	Forward: 5'-GAT GGC TCC CTA CAG AAG AAA C-3'<br>Reverse: 5'-GTT CCT CGA TCT TCA CAC TCT C-3'
S100A8	Forward: 5'-GTG ACA ATG CCG TCT GAA CT-3'<br>Reverse: 5'-GGG CAT GGT GAT TTC CTT GTA-3'
STAT1	Forward: 5'-CTG GAG GAG TTG GAA CAG AAA-3'<br>Reverse: 5'-CTG AAT GAG CTG CTG GAA GA-3'
TLR4	Forward: 5'-GCT TAC ACC ACC TCT CAA ACT-3'<br>Reverse: 5'-ACA GCC ACC AGA TTC TCT AAA C-3'
