# Supplementary material for: Sm-p80-based schistosomiasis vaccine mediated epistatic interactions identified potential immune signatures for vaccine efficacy in mice and baboons
Source: PLoS One. 2017 Feb 13;12(2):e0171677. doi: 10.1371/journal.pone.0171677 (PMC5305113; doi:10.1371/journal.pone.0171677)
Supplement: S7 Fig — (A) Gene ontology analysis of differentially expressed genes (y axis) according to biological functions (legends). Analysis was performed with PANTHER GO Classification System. Differences in number of genes were observed across baboon tissues (x axis). (B) Venn diagrams illustrating genes overlapping across different baboon tissues. Heat maps of genes common across different tissues. Peripheral blood mononuclear cells collected before (week 12) and after (week 20) S. mansoni challenge infection where compared. Fold change expression values observed across different tissue comparisons: (C) PBMC, spleen, and lymph nodes; (D) PBMC at weeks 12 and 20; (E) PBMC and spleen; (F) PBMC and lymph nodes; and (G) spleen and lymph nodes. (PDF) [file pone.0171677.s014.pdf]

A

# S7 Fig

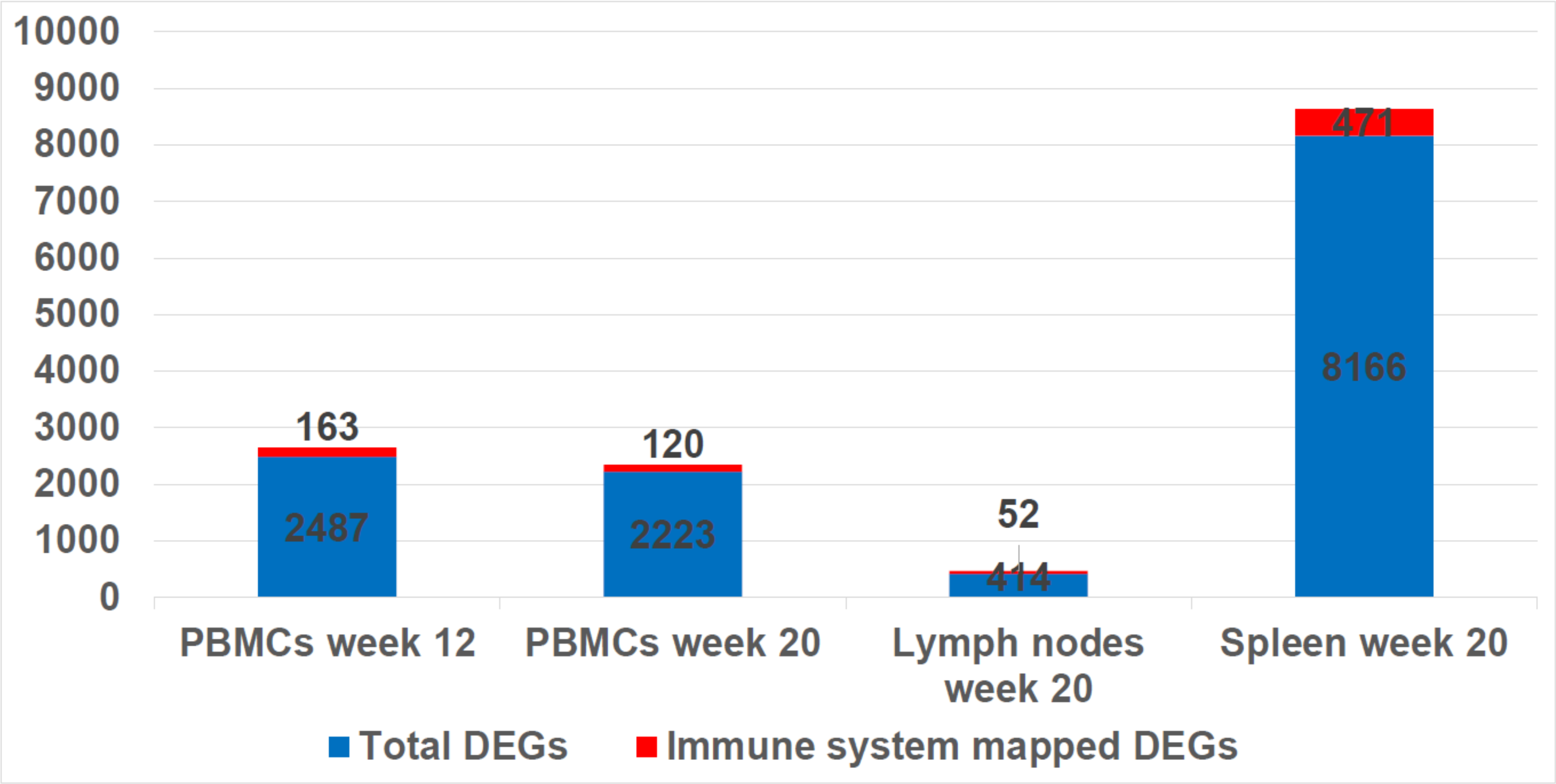

10000
9000
8000
7000
6000
5000
4000
3000
2000
1000
0
163
2487
120
2223
52
414
471
8166
PBMCs week 12
PBMCs week 20
Lymph nodes week 20
Spleen week 20
Total DEGs
Immune system mapped DEGs

**B**

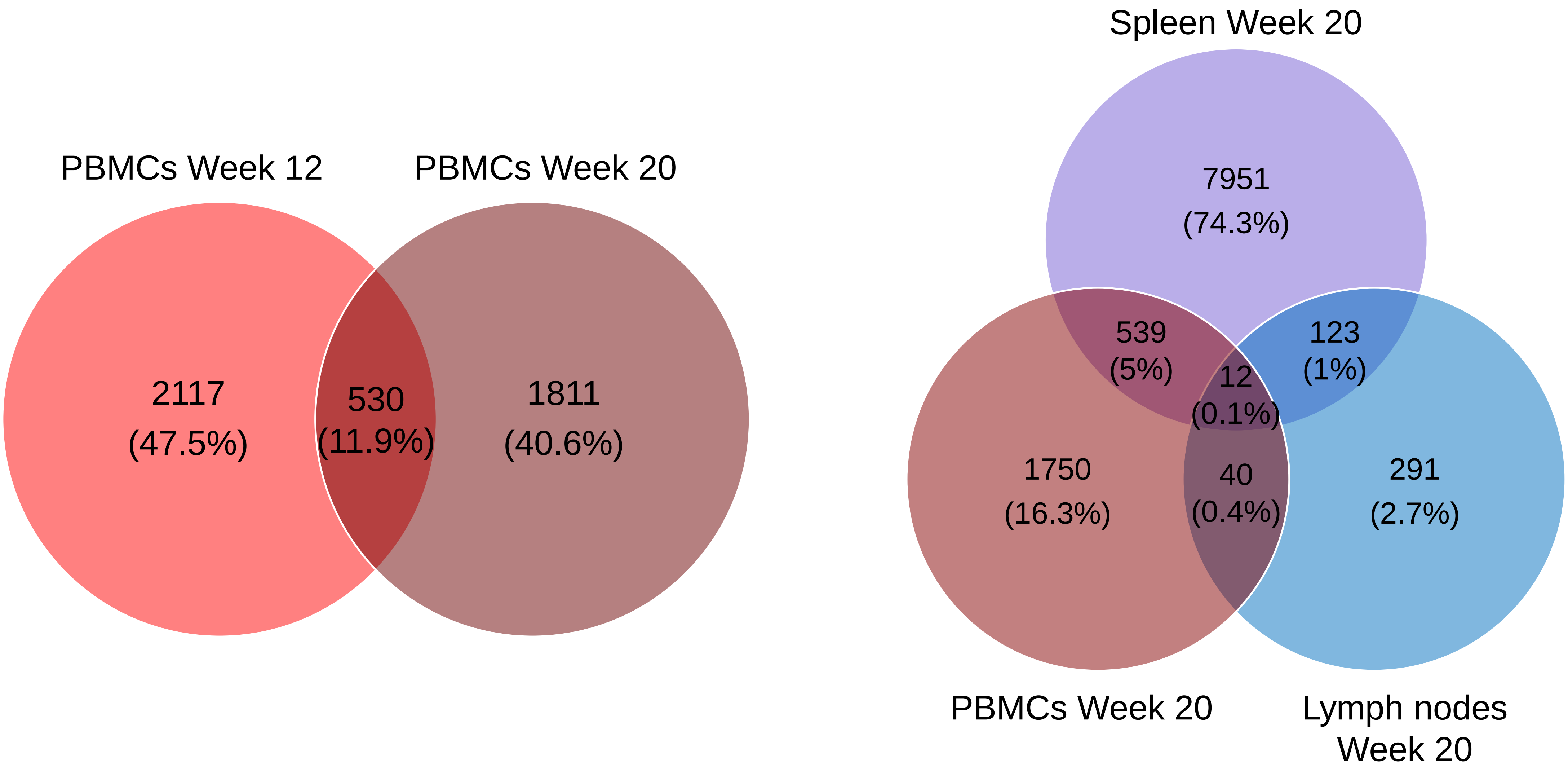
PBMCs Week 12
PBMCs Week 20
2117
(47.5%)
530
(11.9%)
1811
(40.6%)
Spleen Week 20
7951
(74.3%)
539
(5%)
123
(1%)
12
(0.1%)
1750
(16.3%)
40
(0.4%)
291
(2.7%)
PBMCs Week 20
Lymph nodes
Week 20

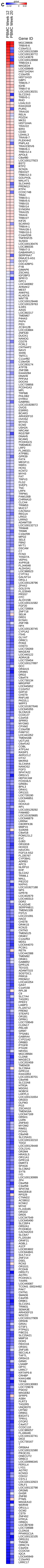
c
global
-11.52
0
8.02
PBMC Week 12
PBMC Week 20
Gene ID
MGC39606
TRBV6-6
C10orf111
LOC100131589
LOC100130772
LOC143678
LOC100128968
TRBV24-1
MAGED4
LOC439949
HIST1H2AH
C10orf35
HIST1H1D
GBP1
TRBV5-7
IGHE
TRBV7-8
LOC100130231
SLC22A3
TRBV5-1
RHO
LGALS13
KIAA1618
PURG
TRAV20
RELN
PDZD4
MKS1
HIST1H4A
TRAV22
IER3
CD3D
LDHAL3
TRAV8-7
PLEKHH2
PNPLA3
TRAV23DV6
TRBV6-1
TRBV12-4
APOL6
C8orf80
LOC100127923
BST2
RTP2
SCN2A
FBXO27
TRBV12-3
GOLPH3L
MALAT1
SLC44A5
PRDM13
RTP4
CCDC74B
TRAC
TRBV3-1
TRBV5-5
TRBV15
TRBV14
SYNGR4
TRAV25
TRAV4
TRBV27
KIF25
SCARA3
TRBV4-1
TRAV26-1
TRBV10-3
C14orf156
TNFRSF25
SUSD4
LOC100130476
LOC388335
TRBV7-3
GABARAPL1
SERPINA7
DNAJC3-AS1
DCST2
CDC42BPG
FYB
GIMAP6
ZSCAN1
IGF2BP3
SPRY3
GPC1
LOC440082
AHNAK2
MEST
ANKFN1
KLHDC6
WNT7B
LOC100129446
LOC100129763
ILDR1
AASS
LOC392217
TMEM67
P4HA3
PKP4
PID1
ZC3H12B
LOC153684
ZNF530
DUOX1
NANOG
CD276
FCRL2
CNTNAP2
NAV3
XKR6
TMTC1
C11orf82
C19orf26
LOC283174
ATP7B
ZNF396
KIAA0284
DNAH9
PPFIA4
DOCK6
LOC728858
PCDHGA3
MPL
TRPM6
PHLDB2
DYRK4
GABRB1
LOC101928072
OVOS
ESRRG
BCAR3
ARHGEF10
OVOS2
MYH15
ECM2
USH2A
NRCAM
RGS7BP
DNAH5
NCAM1
PCDHGC5
TMEM63C
LRP2
FLJ46321
ATP8B1
GPR39
FAT4
MEGF11
NWD1
HCN1
KCNT2
MYH3
FAT
TRPV3
FLG2
SVEP1
TNC
LRP4
TRPM1
FAM135B
CHRNA10
CLCA4
TRIM53
MUC17
CACNA1I
REG1A
LRIG3
LRRN3
ADAMTS9
LOC100132713
TRIM48
TRIM9
C10orf39
MPDZ
SELE
FBN2
MYT1
DSCAM
C7
FCN3
THSD7A
VEPH1
AVPR1B
FLJ44048
ALDH3A1
LOC388523
CYP7A1
GALNT14
OR1L1
LOC100128796
ZFHX2
GOLSYN
FLJ23049
PRSS7
OTC
ALOX15B
LOC100131582
FGF20
LOC728904
ZNF718
SELM
DRD1
KCNA1
ZNF786
HPX
LOC100130745
CYP1A2
ITIH5
GLYAT
OR6C1
PON3
CHST3
LOC728269
MAGEA9
LOC440122
ADRA1B
LOC729454
LOC100127897
DCC
OR4A15
OR4M1
DRD2
C8orf74
LOC730134
MRGPRF
LOC645652
C12orf12
GDF10
CHST9
RNF148
PKD2L1
WIPF3
LOC101927646
LOC645355
SLC6A19
CCDC27
C2orf16
SPRR3
MYOM3
CPXM2
C10orf38
FAM71C
LOC441052
TMEM16B
UGT1A5
C6orf142
COBL
ATP1A4
RASIP1
AKR1CL2
OTX2
MKRN3
SLC44A4
NANOS2
FGF3
SPATA22
OR51V1
HEPACAM
FOXD4L5
GREB1
BPIL3
OR4X1
OR13J1
LOC728190
LOC646851
GJA1
HOXA10
RP1
LOC100129292
FAM46D
LOC101929685
LOC646879
LRRN4
C8ORFK36
MMP15
S100A6
C9orf18
POM121L2
EVC
GJB3
OR10G9
HOXD3
HAVCR1
FGF14-AS2
FLJ35767
CYP39A1
ADRB3
APCS
NLRP10
EHF
SLC2A2
TRIML1
MALL
PIEZO2
NXPH1
LOC101927188
NPS
GPR78
LOC402679
LOC440313
DDR2
PLA2G4E
SERPINB11
TMEM132B
FSTL5
LOC221091
HAO2
CHST8
OR4K15
KCNJ3
CNTN3
TMEM125
OR4E2
C12orf53
MDS1
LOC644070
PCSK1
RETN
LOC442388
TMEM92
OR4K1
GABBR2
GPR125
TEX101
ADAMTS15
SOSTDC1
PRR16
PRDM12
LOC441054
GAST
C1orf158
RPL38
C9
SLC7A4
TAS2R1
PPEF2
LAMP3
FLJ20712
FREM2
STEAP2
OR1B1
OPRK1
LOC729549
CLDN17
PRLHR
GRM7
TPSAB1
GABRA4
CYP21A2
OR6B3
PTGFR
PTH2R
NRXN1
MGC39584
CLDN8
MAGEC3
LOC202459
TMEM28
SLC39A4
GPR110
KIAA1853
LOC100130701
C8orf46
SLC22A8
HTR3A
MSGN1
NOBOX
LHFPL3
LOC100131654
OR2D3
OLFM3
PI15
ZSCAN10
TMEM16A
LOC647169
OR5H1
LGR6
ZNF643
MYOM2
RIMS1
FOXA1
C8orf48
SLC25A31
LOC100132210
TDO2
LOC100129448
COL21A1
OR2M4
HRASLS
OR11A1
GPR116
DMRT3
SPAG6
SLC36A2
SYT6
IYD
LOC100130999
ZP4
C6orf58
C3orf59
SPATA6
MAGEB18
RPS29
CTNND2
ACSBG2
KCNK2
MYH2
FLJ16165
OR1D2
DRD5
LOC347549
LOC645490
SLC35F4
OR52A1
PCDHB10
CACNA2D3
SLC6A7
FLJ20184
OR4F21
AGBL1
A1CF
LOC653602
LOC644841
SULT1C2
ZNF214
DSEL
RCN3
OR10J1
PCDH9
OR51T1
AGTR1
PCDHAC1
TAAR5
LOC440087
TCONS_00024492
PAK7
CNTN1
SMAD6
C4orf35
OR8K1
SLC15A1
TRIM31
SEMA3E
ARHGEF15
WSCD2
LOC100127909
OR5H6
GRIA1
GTSF1
PTPN21
FIBCD1
SLC25A21
MMP16
DOK5
FBXO16
OR3A1
ZNF185
SEC14L4
TAF7L
OR5H15
GRM3
LSAMP
OPTC
LRRC7
KRTAP5-8
CRHBP
KIAA1486
TIFAB
LOC100130083
LOC729678
PSKH2
MAGEB2
SFRP5
ASB4
FLRT3
TAS2R5
UNQ9370
LRTM2
OR8S1
B3GAT1
TPRX1
OTOP2
C4orf17
CCDC110
LOC389257
FLJ46446
LOC100132741
DIO2
LOC730167
T
OR56A4
LOC100131580
PIK3C2G
ONECUT2
OR6C3
LOC100996345
FER1L4
LYG1
AGTR2
KCNS3
CDH12
CSTL1
LOC100132923
CRLF1
LOC100132798
OR2M5
GHSR
ZNF80
PTMS
MAGEA10
WDR49
OR11L1
GCM2
CST9
ZNF452
HTR1A
OR6Q1
LOC387939
LOC728588
CLDN16
PPARGC1A
LOC100132105
OR51S1
TNNI3K
OR6C74
C3orf24
CCDC54
IQCA
LOC645949
C15orf43
LRRC17

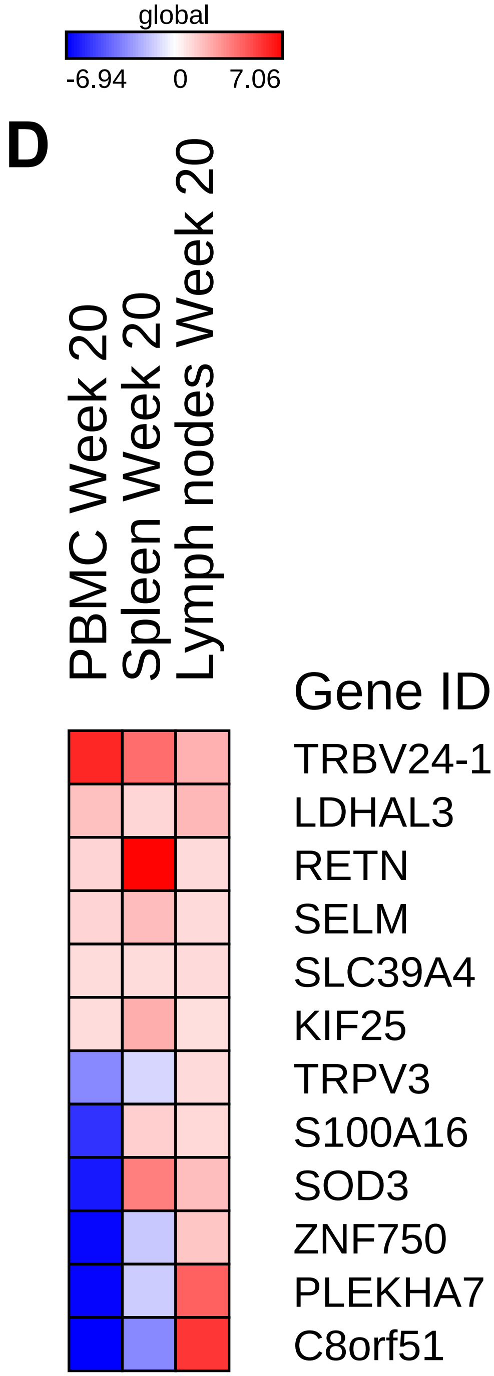
global
-6.94
0
7.06
D
PBMC Week 20
Spleen Week 20
Lymph nodes Week 20
Gene ID
TRBV24-1
LDHAL3
RETN
SELM
SLC39A4
KIF25
TRPV3
S100A16
SOD3
ZNF750
PLEKHA7
C8orf51

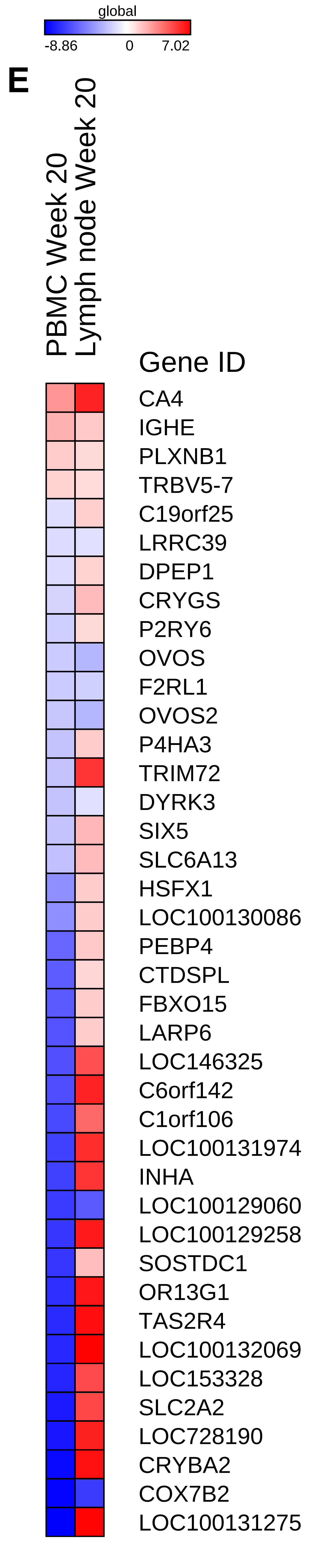
global
-8.86
0
7.02
E
PBMC Week 20
Lymph node Week 20
Gene ID
CA4
IGHE
PLXNB1
TRBV5-7
C19orf25
LRRC39
DPEP1
CRYGS
P2RY6
OVOS
F2RL1
OVOS2
P4HA3
TRIM72
DYRK3
SIX5
SLC6A13
HSFX1
LOC100130086
PEBP4
CTDSPL
FBXO15
LARP6
LOC146325
C6orf142
C1orf106
LOC100131974
INHA
LOC100129060
LOC100129258
SOSTDC1
OR13G1
TAS2R4
LOC100132069
LOC153328
SLC2A2
LOC728190
CRYBA2
COX7B2
LOC100131275

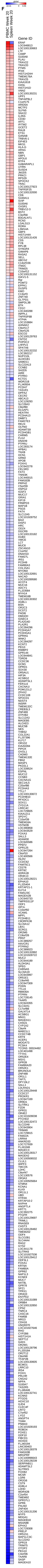
global
-10.31
0
6.56
F
PBMC Week 20
Spleen Week 20
Gene ID
ERAF
LOC646813
LOC100130663
LTF
CAMP
S100A8
PLAU
TRAV22
TRAV20
PTMS
IFI44
HIST1H2AH
TMEM176A
TRAV25
KIAA1618
OASL
HIST1H1D
VNN2
LOC100130231
UPP1
TNFAIP8L2
C1orf175
ARID5A
MCTP2
SLFN5
SNX22
IL2RA
CD3D
AFF1
IFITM2
GCET2
RALB
ETS1
GANAB
TRBV6-1
EIF4E3
MKS1
HLA-G
ARSG
NBN
GBP1
APOL6
RTP4
GABARAPL1
UTRN
SUSD4
JMJD5
PRKCI
RPS29
SPOCK2
TRAC
LOC100127923
TNFRSF25
LOC100132000
CD6
S100A11
SVIP
S100A6
OXSR1
TRBV12-3
BST2
C3orf58
B3GALNT1
RCN3
LGALS13
RORA
LSM14A
GBP4
HIST1H4A
LOC100131426
CD5
FYB
RPL38
SYNGR4
GIMAP6
RASA2
SELL
ABCG2
C14orf156
ITM2C
TRIM25
C10orf12
LOC100131152
IGKV1-5
Rgr
POMT2
EPPK1
DUSP5
KMO
C13orf3
ZNF740
GLTPD1
SMPDL3B
BTK
CD1C
LOC440288
INPPL1
TNFRSF6B
ATP4A
LOC153684
AHNAK2
C9orf117
C9orf125
ESCO2
LOC100129763
CNTD2
POPDC2
LHFPL5
SPATA6
LOC100132232
LOC392217
NUP210L
ALDH1B1
SAMD11
SLC22A13
CCNB2
SH2D5
AIRE
PTPRO
KCTD1
WDR21B
FLJ40504
PCDH7
TAS1R1
DPPA2
CECR2
ABCA13
LOC129293
SLC26A3
C8orf80
DLGAP1
HEATR4
PCDHA13
DMBT1
FAM27E3
RELN
OLFM2
ADAMTS9
PCDHGB1
CHRM5
FLG2
DNAH9
PTPRU
LOC283174
APBA1
TNXB
HCN1
GRM2
APOB
FOXH1
LOC642278
CELSR3
FAT3
TNNI3K
LOC440015
FAM182B
PRPH2
C5orf39
HSPG2
IGSF3
MUC17
GRIA1
KIF19
FRMPD3
MAP1B
PSG5
FAT4
SLC12A5
LOC101928752
BAI2
DISP2
HRC
ODZ4
DGCR9
LOC100133182
DUB3
VWCE
LCT
MUC6
ZSCAN10
C1orf32
DNAH10
FAM27L
IRS1
PCDH9
FAM90A3
COL15A1
MYOM2
LOC100132691
NACAD
LOC100289580
ACOT4
MUC16
ADCY1
FLJ23584
MYH14
TMEM16B
LOC100130302
AGBL1
BSN
OR2C3
DCST2
PADI6
DMGDH
IQSEC3
PLA2G4D
PLA2R1
LOC284296
PCDHGA7
PCDHGA1
GPRIN1
WNK2
LOC284297
KCNT2
PEG3
RIMBP2
GPR144
LOC644841
TDRD9
CCDC36
GCM1
OPRD1
SOX30
OR5AU1
HIF3A
ACSBG2
ADAMTSL1
FER1L6
CACNA1I
POM121L2
ZFHX2
CCDC108
SPOCD1
NDST4
INSRR
TMEM132C
CREB3L3
ZNF469
ATP2B2
SLC12A3
MAGEB5
ALOXE3
MUC5B
NES
THBS2
COL22A1
FLJ37396
KLF17
OR1D2
KIAA0284
PIPOX
OVOL1
CADM3
TMEM132E
FBN2
MASP1
PKD2L1
LOC339524
ESPNL
MUC12
CCNB3
CACNA1G
SEC14L5
C20orf86
PCDHA3
TTLL7
LOC100130672
PCDHB10
RFXDC1
SOX11
ZYG11A
LRRC4C
LOC729605
KIAA1211
SPDYC
C10orf39
TMEM16A
VSTM2B
LOC390858
LOC643529
FOXA3
SALL3
ADAM30
LOC645586
PPEF2
GPR125
LOC647264
C6orf10
LOC400566
ISLR2
CCDC62
FAM75C1
CD276
KRT6C
ADRA1B
OR4K13
LOC100128221
EPHA10
FLJ25404
KRTAP21-1
KLF14
FAM123C
MAGEA2B
LOC645733
TMPRSS11F
ODF1
AIF1L
C10orf92
VCAM1
MPFL
PCDHB11
C8ORFK36
SLC6A12
LBX1
SCRG1
OR2M5
C19orf26
PRNT
LOC389257
HAVCR1
PTPRN2
LOC389023
LOC100132892
LOC101928722
MUC2
LOC644189
ALDH3A1
SPEG
CHRNA5
SLC6A19
LOC643997
OR51E1
C19orf45
LOC647309
PODN
TTLL6
RBM35A
PTPRN
LOC728145
TAAR5
LOC643355
SLC32A1
C17orf88
GALNT14
ACSBG1
MNX1
MAGEA11
CASQ1
CYP1A2
C9orf4
MAB21L1
CCDC74B
KRT9
ACER1
MOGAT3
PCSK4
TCONS_00024492
LOC401498
TTYH1
OR52E4
MSX1
GPR39
FAM90A20
OR52K1
BRUNOL6
ZNF488
OR1L1
TAF7L
DPY19L2
SALL1
LDLRAD1
LOC100129448
CCDC27
PROKR1
LOC647169
OR9Q1
SLITRK1
FZD9
GPR31
OR4E2
LOC101928038
PTCHD2
LOC100132472
SLC22A8
LOC643904
TRIM49
LOC729915
FAM22A
LRRN4
ANKRD33
LOC727939
FLJ41046
MAGEC3
LOC100128317
MAGEA2
LOC100506532
CLUL1
WIPF3
TMCO5
LDHC
C8orf47
LOC130576
OR3A3
LOC100505664
STMN4
KCNA1
KLK9
OR11L1
UBD
HTR3A
KRTAP10-2
NXPH4
ANGPTL4
KRT71
LOC392275
PTGFR
LOC100129697
TRIML1
RNASE9
CHST3
LOC100128482
TMEM202
ODF4
SLCO2B1
SLC44A4
RAG2
GYS2
LOC441178
SLITRK6
LOC101927536
LOC100129412
ODF3L1
FOXA1
KRT6A
FAM80A
GPR62
NAP1L3
KCNK9
MUC7
NAT8L
GPR78
SPZ1
TPRX1
OR4Q3
ENTPD2
LOC100131089
OR8B4
LOC100132850
CPN2
TNRC4
CASC16
FAM5B
NRG3
CDH16
UNQ9370
LOC101927646
RHO
CYP2B6
HIST1H1A
SEPT12
GAD1
OR2W1
LOC100128796
FLJ20184
OR2T12
C8orf48
TFAP2B
LOC100130605
BCMO1
LRRC10
IRS4
FOXI1
PRLHR
CNGA2
NPPA
S100A7
TAS2R5
FLJ46446
LOC100132741
KCNN3
PTGDS
HOXC10
GJD4
CLEC4F
LRIT2
C7orf52
LYG2
ANGPT4
TGM4
PNMA3
LOC101928193
ROBO4
FOXE1
GDF10
FIBCD1
HOXD3
FLJ40194
LINC00403
LOC100128378
FAM163B
MRGPRF
LOC100996643
LOC100128239
SERPINB11
ADAMTSL2
SLITRK5
FOXB2
MC5R
LGR6
OR2G6
CST9
AGBL4
LDHD
WDR42B
C2orf54
TMEM92
GPR37L1
GPR6
PALM2
ZNF404
LOC100131206
MYF5
MDGA1
MAGEB18
MS4A8B
EPHX2
LOC729308
PRELP
OR51S1
MAP1LC3C
SLC22A3
GPR19
TRIM75
OTOP2
PEG10
C4orf36
TAAR8
SASP
MAGEA10

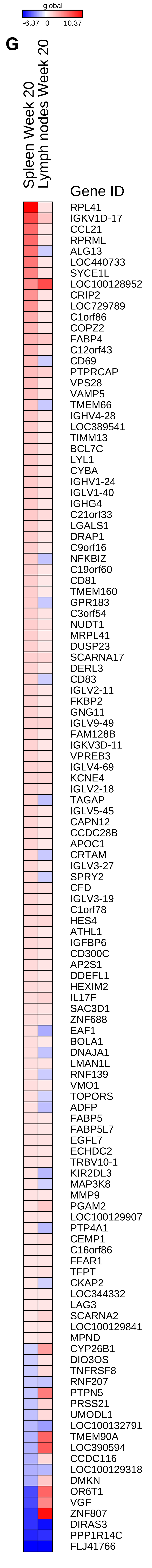
G
global
-6.37 0 10.37
Spleen Week 20
Lymph nodes Week 20
Gene ID
RPL41
IGKV1D-17
CCL21
RPRML
ALG13
LOC440733
SYCE1L
LOC100128952
CRIP2
LOC729789
C1orf86
COPZ2
FABP4
C12orf43
CD69
PTPRCAP
VPS28
VAMP5
TMEM66
IGHV4-28
LOC389541
TIMM13
BCL7C
LYL1
CYBA
IGHV1-24
IGLV1-40
IGHG4
C21orf33
LGALS1
DRAP1
C9orf16
NFKBIZ
C19orf60
CD81
TMEM160
GPR183
C3orf54
NUDT1
MRPL41
DUSP23
SCARNA17
DERL3
CD83
IGLV2-11
FKBP2
GNG11
IGLV9-49
FAM128B
IGKV3D-11
VPREB3
IGLV4-69
KCNE4
IGLV2-18
TAGAP
IGLV5-45
CAPN12
CCDC28B
APOC1
CRTAM
IGLV3-27
SPRY2
CFD
IGLV3-19
C1orf78
HES4
ATHL1
IGFBP6
CD300C
AP2S1
DDEFL1
HEXIM2
IL17F
SAC3D1
ZNF688
EAF1
BOLA1
DNAJA1
LMAN1L
RNF139
VMO1
TOPORS
ADFP
FABP5
FABP5L7
EGFL7
ECHDC2
TRBV10-1
KIR2DL3
MAP3K8
MMP9
PGAM2
LOC100129907
PTP4A1
CEMP1
C16orf86
FFAR1
TFPT
CKAP2
LOC344332
LAG3
SCARNA2
LOC100129841
MPND
CYP26B1
DIO3OS
TNFRSF8
RNF207
PTPN5
PRSS21
UMODL1
LOC100132791
TMEM90A
LOC390594
CCDC116
LOC100129318
DMKN
OR6T1
VGF
ZNF807
DIRAS3
PPP1R14C
FLJ41766
