# Supplementary material for: Sm-p80-based schistosomiasis vaccine mediated epistatic interactions identified potential immune signatures for vaccine efficacy in mice and baboons
Source: PLoS One. 2017 Feb 13;12(2):e0171677. doi: 10.1371/journal.pone.0171677 (PMC5305113; doi:10.1371/journal.pone.0171677)
Supplement: S1 Table — Red: S. mansoni control challenge; Yellow: DNA vaccine (VR1020-Sm-p80); green: prime/boost (pcDNA3 –Sm-p80 + rSm-p80 + ODN); aqua: rSm-p80 + alum; blue: rSm-p80 + GLA; and violet: rSm-p80 + ODN. The symbol “X” indicates identified genes in dataset overlapping with the corresponding canonical pathway. (PDF) [file pone.0171677.s016.pdf]

S1 Table

Number	Canonica pathway	S. mansoni control challenge	VR1020-Sm-p80	pcDNA3-Sm-p80 + rSm-p80 + ODN	rSm-p80 + alum	rSm-p80 + GLA	rSm-p80 + ODN
1	14-3-3-mediated Signaling	X	X	X			X
2	3-phosphoinositide Biosynthesis	X					X
3	3-phosphoinositide Degradation	X					X
4	4-1BB Signaling in T Lymphocytes	X	X				
5	4-aminobutyrate Degradation I					X	
6	Actin Cytoskeleton Signaling		X	X			X
7	Actin Nucleation by ARP-WASP Complex	X	X				X
8	Activation of IRF by Cytosolic Pattern Recognition Receptors	X					X
9	Acute Myeloid Leukemia Signaling	X	X				X
10	Acute Phase Response Signaling	X					
11	Acyl Carrier Protein Metabolism				X		
12	Adipogenesis pathway						X
13	Agrin Interactions at Neuromuscular Junction		X				X
14	Aldosterone Signaling in Epithelial Cells	X					X
15	Allograft Rejection Signaling		X				
16	Altered T Cell and B Cell Signaling in Rheumatoid Arthritis	X	X				
17	AMPK Signaling	X		X			X
18	Amyloid Processing		X				
19	Amyotrophic Lateral Sclerosis Signaling			X			X
20	Androgen Signaling	X	X				X
21	Angiopoietin Signaling	X					X
22	Antigen Presentation Pathway	X	X				X
23	Antioxidant Action of Vitamin C	X					
24	Antiproliferative Role of Somatostatin Receptor 2						X
25	Antiproliferative Role of TOB in T Cell Signaling	X					
26	Apoptosis Signaling	X	X				X
27	April Mediated Signaling	X					X
28	Arginine Degradation VI (Arginase 2 Pathway)	X					
29	Arsenate Detoxification I (Glutaredoxin)		X				
30	Aryl Hydrocarbon Receptor Signaling	X	X				
31	Assembly of RNA Polymerase I Complex	X					X
32	Assembly of RNA Polymerase II Complex	X	X				
33	Assembly of RNA Polymerase III Complex	X		X			X
34	ATM Signaling	X					
35	Autoimmune Thyroid Disease Signaling	X	X				
36	autophagy		X				X
37	Axonal Guidance Signaling		X	X			X
38	B Cell Activating Factor Signaling	X					
39	B Cell Development	X	X				
40	B Cell Receptor Signaling	X	X	X			X
41	Basal Cell Carcinoma Signaling			X			
42	Biotin-carboxyl Carrier Protein Assembly			X			
43	Bladder Cancer Signaling		X				
44	BMP signaling pathway		X				
45	Breast Cancer Regulation by Stathmin1	X	X				X
46	Calcium Signaling			X		X	X
47	Calcium-induced T Lymphocyte Apoptosis	X	X				
48	cAMP-mediated signaling	X		X	X		X
49	Cardiac Hypertrophy Signaling	X	X				X
50	Cardiac β-adrenergic Signaling		X				X
51	Caveolar-mediated Endocytosis Signaling	X	X	X			X
52	CCR3 Signaling in Eosinophils	X	X				X
53	CCR5 Signaling in Macrophages		X				
54	CD27 Signaling in Lymphocytes	X					X
55	CD28 Signaling in T Helper Cells	X	X				X
56	CD40 Signaling	X	X				X
57	Cdc42 Signaling	X	X				X
58	CDK5 Signaling	X	X				X
59	CDP-diacylglycerol Biosynthesis I		X				
60	Cell Cycle Control of Chromosomal Replication	X	X				
61	Cell Cycle Regulation by BTG Family Proteins	X					
62	Cell Cycle: G1/S Checkpoint Regulation	X	X				X
63	Cell Cycle: G1/S Checkpoint Regulation	X					X
64	Ceramide Signaling	X	X				
65	Chemokine Signaling	X	X				X
66	Cholecystokinin/Gastrin-mediated Signaling	X	X				X
67	Cholesterol Biosynthesis I	X					
68	Cholesterol Biosynthesis II (via 24,25-dihydrolanosterol)	X					
69	Cholesterol Biosynthesis III (via Desmosterol)	X					
70	Chronic Myeloid Leukemia Signaling	X	X				X
71	Circadian Rhythm Signaling						X
72	Clathrin-mediated Endocytosis Signaling						X
73	CNTF Signaling	X	X				X
74	Coagulation System			X			
75	Coenzyme A Biosynthesis		X				
76	Colorectal Cancer Metastasis Signaling	X	X				X
77	Communication between Innate and Adaptive Immune Cells		X				
78	Corticotropin Releasing Hormone Signaling	X		X			X
79	CREB Signaling in Neurons	X	X	X			X
80	Crosstalk between Dendritic Cells and Natural Killer Cells	X	X				X
81	CTLA4 Signaling in Cytotoxic T Lymphocytes	X	X				X
82	CXCR4 Signaling	X	X				X
83	Cyclins and Cell Cycle Regulation	X	X				
84	Cysteine Biosynthesis III (mammalia)	X					
85	Cytotoxic T Lymphocyte-mediated Apoptosis of Target Cells	X	X			X	
86	Death Receptor Signaling	X	X				X
87	Dendritic Cell Maturation	X	X				X
88	Diphthamide Biosynthesis		X				
89	D-myo-inositol (1,3,4)-trisphosphate Biosynthesis			X			X
90	D-myo-inositol (1,4,5)-trisphosphate Degradation	X		X			X
91	D-myo-inositol (3,4,5,6)-tetrakisphosphate Biosynthesis	X					X
92	D-myo-inositol-5-phosphate Metabolism	X					X
93	DNA damage-induced 14-3-3σ Signaling	X					

94	DNA Double-Strand Break Repair by Homologous Recombination	X					
95	DNA Double-Strand Break Repair by Non-Homologous End Joining	X					X
96	DNA Methylation and Transcriptional Repression Signaling	X					X
97	Docosahexaenoic Acid (DHA) Signaling						X
98	Dopamine Receptor Signaling		X				
99	Dopamine-DARPP32 Feedback in cAMP Signaling	X	X	X			X
100	EGF Signaling	X		X			X
101	EIF2 Signaling				X	X	X
102	Endometrial Cancer Signaling	X	X	X			X
103	Endoplasmic Reticulum Stress Pathway	X					X
104	Endothelin-1 Signaling	X	X	X			X
105	eNOS Signaling			X			
106	Ephrin A Signaling			X			X
107	Ephrin B Signaling	X	X				X
108	Ephrin Receptor Signaling	X	X				X
109	Epithelial Adherens Junction Signaling			X			X
110	ErbB Signaling	X	X				X
111	ErbB2-ErbB3 Signaling	X	X				
112	ErbB4 Signaling	X	X	X			X
113	ERK/MAPK Signaling	X	X				X
114	ERK5 Signaling	X					X
115	Erythropoietin Signaling	X	X	X			X
116	Estrogen Receptor Signaling	X	X	X			X
117	Estrogen-Dependent Breast Cancer Signaling	X					X
118	Estrogen-mediated S-phase Entry	X					
119	Factors Promoting Cardiogenesis in Vertebrates			X			X
120	FAK Signaling	X	X	X			X
121	Fatty Acid Activation			X			
122	Fatty Acid β-oxidation I		X				
123	Fc Epsilon RI Signaling	X	X				
124	Fcγ Receptor-mediated Phagocytosis in Macrophages and Monocytes	X	X				X
125	FcγRIIB Signaling in B Lymphocytes	X	X				
126	FGF Signaling			X			
127	FLT3 Signaling in Hematopoietic Progenitor Cells	X	X	X			X
128	fMLP Signaling in Neutrophils	X	X				X
129	Folate Polyglutamylation	X					
130	Folate Transformations I	X					
131	G Beta Gamma Signaling	X	X				X
132	G Protein Signaling Mediated by Tubby		X				X
133	GABA Receptor Signaling						X
134	GADD45 Signaling	X	X				
135	Gap Junction Signaling	X	X	X			X
136	GDNF Family Ligand-Receptor Interactions	X	X				X
137	Geranylgeranyldiphosphate Biosynthesis			X			
138	Germ Cell-Sertoli Cell Junction Signaling	X	X	X			X
139	Glioblastoma Multiforme Signaling	X	X	X			X
140	Glioma Invasiveness Signaling		X	X			X
141	Glioma Signaling	X	X	X			X
142	Glucocorticoid Receptor Signaling	X	X				X
143	Gluconeogenesis I		X			X	
144	Glutamate Degradation III (via 4-aminobutyrate)					X	
145	Glutamate Receptor Signaling			X			X
146	Glutaryl-CoA Degradation	X	X				
147	Glycogen Degradation III		X				
148	Glycolysis I		X				
149	GM-CSF Signaling	X	X				X
150	GNRH Signaling	X	X	X			X
151	GPCR-Mediated Integration of Enteroendocrine Signaling Exemplified by an L Cell						X
152	GPCR-Mediated Nutrient Sensing in Enteroendocrine Cells			X			X
153	G-Protein Coupled Receptor Signaling	X		X			X
154	Graft-versus-Host Disease Signaling	X	X				
155	Granzyme B Signaling	X					X
156	Growth Hormone Signaling	X		X			
157	Gα12/13 Signaling	X	X	X			X
158	Gαi Signaling						X
159	Gαq Signaling	X	X				X
160	Gαs Signaling			X			X
161	Heme Degradation		X				
162	Hepatic Fibrosis / Hepatic Stellate Cell Activation			X			X
163	HER-2 Signaling in Breast Cancer	X					X
164	Hereditary Breast Cancer Signaling	X	X				
165	HGF Signaling	X		X			X
166	HIF1α Signaling		X				
167	HIPPO signaling	X					X
168	HMGB1 Signaling	X	X				
169	Human Embryonic Stem Cell Pluripotency			X			
170	Huntington's Disease Signaling	X					X
171	Hypoxia Signaling in the Cardiovascular System	X					X
172	iCOS-iCOSL Signaling in T Helper Cells	X	X				X
173	IGF-1 Signaling	X		X			X
174	IL-1 Signaling	X					X
175	IL-10 Signaling	X					
176	IL-12 Signaling and Production in Macrophages	X					
177	IL-15 Production	X					
178	IL-15 Signaling	X	X				X
179	IL-17 Signaling	X					
180	IL-17A Signaling in Airway Cells	X					X
181	IL-17A Signaling in Fibroblasts		X				X
182	IL-17A Signaling in Gastric Cells		X				
183	IL-2 Signaling	X	X				X
184	IL-22 Signaling	X					
185	IL-3 Signaling	X	X				X
186	IL-4 Signaling	X	X	X			X
187	IL-6 Signaling	X	X				
188	IL-8 Signaling	X	X				X
189	IL-9 Signaling	X					
190	ILK Signaling	X	X			X	X
191	Induction of Apoptosis by HIV1	X	X				

		Grey	Blue	Yellow	Green	Purple	Red
192	Inhibition of Angiogenesis by TSP1	X	X				
193	iNOS Signaling	X					X
194	Inosine-5'-phosphate Biosynthesis II	X					
195	Inositol Pyrophosphates Biosynthesis			X			
196	Insulin Receptor Signaling	X	X	X			X
197	Integrin Signaling	X	X				X
198	Interferon Signaling	X	X				X
199	Intrinsic Prothrombin Activation Pathway			X			
200	Isoleucine Degradation I		X				
201	JAK/Stat Signaling	X	X				X
202	Ketogenesis	X	X				
203	Ketolysis	X	X				
204	L-DOPA Degradation		X				
205	Leptin Signaling in Obesity	X		X			X
206	Leucine Degradation I	X	X				
207	Leukocyte Extravasation Signaling	X		X			X
208	Lipid Antigen Presentation by CD1						
209	LPS-stimulated MAPK Signaling	X	X				X
210	Lymphotoxin β Receptor Signaling	X	X				X
211	Macropinocytosis Signaling	X	X				
212	Mechanisms of Viral Exit from Host Cells	X					
213	Melanocyte Development and Pigmentation Signaling	X		X			X
214	Melanoma Signaling	X	X				
215	Melatonin Degradation III				X		
216	Melatonin Signaling						X
217	Methionine Degradation I (to Homocysteine)	X		X			
218	Mevalonate Pathway I	X					
219	MIF Regulation of Innate Immunity	X	X				
220	MIF-mediated Glucocorticoid Regulation	X					
221	Mismatch Repair in Eukaryotes	X					X
222	Mitochondrial Dysfunction				X		X
223	Mitochondrial L-carnitine Shuttle Pathway			X			
224	Mitotic Roles of Polo-Like Kinase	X	X				
225	Molecular Mechanisms of Cancer	X	X	X			X
226	Molybdenum Cofactor Biosynthesis	X					
227	Mouse Embryonic Stem Cell Pluripotency	X	X	X			X
228	MSP-RON Signaling Pathway			X			X
229	mTOR Signaling						X
230	Myc Mediated Apoptosis Signaling	X	X				X
231	NADH Repair		X				
232	Natural Killer Cell Signaling	X	X				X
233	Netrin Signaling						X
234	Neuregulin Signaling	X					X
235	Neuropathic Pain Signaling In Dorsal Horn Neurons			X			X
236	Neuroprotective Role of THOP1 in Alzheimer's Disease		X				
237	Neurotrophin/TRK Signaling	X		X			X
238	NF-κB Activation by Viruses	X	X	X			X
239	NF-κB Signaling	X		X			X
240	NGF Signaling	X	X	X			X
241	Nitric Oxide Signaling in the Cardiovascular System			X			
242	nNOS Signaling in Skeletal Muscle Cells			X			
243	Non-Small Cell Lung Cancer Signaling	X	X				X
244	Notch Signaling	X		X			X
245	NRF2-mediated Oxidative Stress Response	X	X				X
246	Nucleotide Excision Repair Pathway	X					
247	Nur77 Signaling in T Lymphocytes	X	X				
248	Oncostatin M Signaling	X	X				
249	Ovarian Cancer Signaling	X		X			
250	OX40 Signaling Pathway		X				
251	Oxidative Phosphorylation				X		X
252	P2Y Purigenic Receptor Signaling Pathway	X	X				X
253	p38 MAPK Signaling	X	X				
254	p53 Signaling	X					X
255	p70S6K Signaling	X	X	X			X
256	PAK Signaling	X	X				X
257	Pancreatic Adenocarcinoma Signaling	X	X				X
258	Paxillin Signaling	X	X	X			X
259	PDGF Signaling	X	X	X			X
260	PEDF Signaling	X	X				
261	phagosome formation			X			X
262	phagosome maturation		X				X
263	Phosphatidylglycerol Biosynthesis II (Non-plastidic)		X				
264	Phospholipase C Signaling	X	X				X
265	PI3K Signaling in B Lymphocytes	X	X	X			X
266	PI3K/AKT Signaling	X					X
267	PKCθ Signaling in T Lymphocytes	X	X				X
268	PPAR Signaling						X
269	PPARα/RXRα Activation	X		X			X
270	Primary Immunodeficiency Signaling	X	X				
271	Production of Nitric Oxide and Reactive Oxygen Species in Macrophages	X	X				X
272	Prolactin Signaling	X	X	X			X
273	Proline Biosynthesis I	X					
274	Proline Biosynthesis II (from Arginine)	X					
275	Prostate Cancer Signaling	X				X	X
276	Protein Kinase A Signaling	X	X	X			X
277	Protein Ubiquitination Pathway	X			X		X
278	PRPP Biosynthesis I						
279	PTEN Signaling			X			X
280	Pyridoxal 5'-phosphate Salvage Pathway	X					
281	Pyrimidine Ribonucleotides De Novo Biosynthesis		X				
282	Rac Signaling	X	X				X
283	RAN Signaling						X
284	RANK Signaling in Osteoclasts	X		X			X
285	RAR Activation	X		X			X
286	Reelin Signaling in Neurons	X					X
287	Regulation of Actin-based Motility by Rho		X				X
288	Regulation of Cellular Mechanics by Calpain Protease	X	X				X
289	Regulation of eIF4 and p70S6K Signaling	X					X

290	Regulation of IL-2 Expression in Activated and Anergic T Lymphocytes	X	X				X
291	Regulation of the Epithelial-Mesenchymal Transition Pathway	X		X			X
292	Relaxin Signaling	X					X
293	Remodeling of Epithelial Adherens Junctions	X	X	X	X		X
294	Renal Cell Carcinoma Signaling	X	X				X
295	Renin-Angiotensin Signaling	X	X	X			X
296	Retinoic acid Mediated Apoptosis Signaling	X					
297	RhoA Signaling		X				X
298	RhoGDI Signaling		X				X
299	Role of BRCA1 in DNA Damage Response	X	X				X
300	Role of CHK Proteins in Cell Cycle Checkpoint Control	X					
301	Role of IL-17A in Arthritis	X					
302	Role of JAK family kinases in IL-6-type Cytokine Signaling	X					
303	Role of JAK1 and JAK3 in γc Cytokine Signaling	X	X				X
304	Role of JAK2 in Hormone-like Cytokine Signaling	X					
305	Role of Macrophages, Fibroblasts and Endothelial Cells in Rheumatoid Arthr	X					X
306	Role of MAPK Signaling in the Pathogenesis of Influenza	X	X				
307	Role of NANOG in Mammalian Embryonic Stem Cell Pluripotency			X			X
308	Role of NFAT in Cardiac Hypertrophy	X	X				X
309	Role of NFAT in Regulation of the Immune Response	X	X				X
310	Role of Oct4 in Mammalian Embryonic Stem Cell Pluripotency						X
311	Role of Osteoblasts, Osteoclasts and Chondrocytes in Rheumatoid Arthritis	X		X			X
312	Role of p14/p19ARF in Tumor Suppression	X					X
313	Role of PI3K/AKT Signaling in the Pathogenesis of Influenza	X					X
314	Role of PKR in Interferon Induction and Antiviral Response	X					
315	Role of RIG1-like Receptors in Antiviral Innate Immunity						
316	Role of Tissue Factor in Cancer	X	X				
317	Role of Wnt/GSK-3β Signaling in the Pathogenesis of Influenza			X			
318	Salvage Pathways of Pyrimidine Ribonucleotides	X	X				
319	SAPK/JNK Signaling	X	X				X
320	Selenocysteine Biosynthesis II (Archaea and Eukaryotes)		X				
321	Semaphorin Signaling in Neurons						X
322	Serine Biosynthesis	X					
323	Sertoli Cell-Sertoli Cell Junction Signaling		X	X			X
324	Signaling by Rho Family GTPases		X				X
325	Small Cell Lung Cancer Signaling	X					X
326	Sonic Hedgehog Signaling					X	
327	Sperm Motility			X			
328	Spermidine Biosynthesis I	X					
329	Spermine Biosynthesis	X					
330	Sphingosine-1-phosphate Signaling	X	X				X
331	STAT3 Pathway	X		X			X
332	Sucrose Degradation V (Mammalian)		X				
333	Superpathway of Cholesterol Biosynthesis	X					
334	Superpathway of D-myo-inositol (1,4,5)-trisphosphate Metabolism			X			
335	Superpathway of Inositol Phosphate Compounds			X			X
336	Superpathway of Methionine Degradation	X					
337	Superpathway of Serine and Glycine Biosynthesis I	X					
338	Synaptic Long Term Depression	X		X			X
339	Synaptic Long Term Potentiation	X	X	X			X
340	Systemic Lupus Erythematosus Signaling		X				
341	T Cell Receptor Signaling	X	X				X
342	T Helper Cell Differentiation	X	X				
343	TCA Cycle II (Eukaryotic)	X	X				
344	Tec Kinase Signaling	X	X				X
345	Telomerase Signaling	X	X				
346	Tetrahydrobiopterin Biosynthesis I		X				
347	Tetrahydrobiopterin Biosynthesis II		X				
348	TGF-β Signaling		X				X
349	Thio-molybdenum Cofactor Biosynthesis				X		
350	Thiosulfate Disproportionation III (Rhodanese)		X				
351	Thrombin Signaling	X	X	X			X
352	Thrombopoietin Signaling	X	X	X			X
353	Thymine Degradation			X			
354	Thyroid Cancer Signaling	X					
355	Tight Junction Signaling						X
356	TNFR1 Signaling	X					
357	TNFR2 Signaling	X					X
358	Toll-like Receptor Signaling	X					
359	TR/RXR Activation			X			X
360	TREM1 Signaling	X					X
361	Triacylglycerol Degradation		X				
362	tRNA Charging	X					
363	Tryptophan Degradation III (Eukaryotic)	X	X				
364	Tumoricidal Function of Hepatic Natural Killer Cells	X					
365	TWEAK Signaling	X					
366	Type I Diabetes Mellitus Signaling	X	X				
367	Type II Diabetes Mellitus Signaling	X		X			
368	UDP-N-acetyl-D-galactosamine Biosynthesis II	X	X				
369	Unfolded protein response	X					
370	Uracil Degradation II (Reductive)			X			
371	Uridine-5'-phosphate Biosynthesis		X				
372	UVA-Induced MAPK Signaling	X	X	X			X
373	UVB-Induced MAPK Signaling	X					
374	UVC-Induced MAPK Signaling	X	X				
375	Valine Degradation I	X	X				
376	VDR/RXR Activation						X
377	VEGF Family Ligand-Receptor Interactions	X	X	X			
378	VEGF Signaling	X				X	X
379	Virus Entry via Endocytic Pathways	X	X				X
380	Wnt/Ca+ pathway			X			X
381	Wnt/β-catenin Signaling	X					X
382	Xenobiotic Metabolism Signaling						X
383	Zymosterol Biosynthesis	X					
384	α-Adrenergic Signaling		X				X
385	γ-glutamyl Cycle		X				
