# Supplementary material for: Sm-p80-based schistosomiasis vaccine mediated epistatic interactions identified potential immune signatures for vaccine efficacy in mice and baboons
Source: PLoS One. 2017 Feb 13;12(2):e0171677. doi: 10.1371/journal.pone.0171677 (PMC5305113; doi:10.1371/journal.pone.0171677)
Supplement: S2 Table — Lavender: peripheral blood mononuclear cells at week 12 (immunized); purple: peripheral blood mononuclear cells at week 20 (immunized and S. mansoni infected); red: spleen; and blue: lymph nodes. The symbol “X” indicates genes in dataset overlapping with the corresponding canonical pathway. (PDF) [file pone.0171677.s017.pdf]

S2 Table

Number	Canonical pathway	PBMC Week 12	PBMC Week 20	Lymph nodes Week 20	Spleen Week 20
1	14-3-3-mediated Signaling				X
2	3-phosphoinositide Biosynthesis				X
3	3-phosphoinositide Degradation			X	X
4	4-1BB Signaling in T Lymphocytes				X
5	Acetone Degradation I (to Methylglyoxal)	X			
6	Actin Cytoskeleton Signaling				X
7	Actin Nucleation by ARP-WASP Complex				X
8	Activation of IRF by Cytosolic Pattern Recognition Receptors				X
9	Acute Myeloid Leukemia Signaling				X
10	Acute Phase Response Signaling				X
11	Adipogenesis pathway				X
12	Agranulocyte Adhesion and Diapedesis	X	X		
13	Aldosterone Signaling in Epithelial Cells			X	X
14	AMPK Signaling				X
15	Amyloid Processing				X
16	Androgen Signaling				X
17	Angiopoietin Signaling				X
18	Antigen Presentation Pathway				X
19	Antiproliferative Role of Somatostatin Receptor 2				X
20	Antiproliferative Role of TOB in T Cell Signaling				X
21	Apoptosis Signaling				X
22	April Mediated Signaling				X
23	Aryl Hydrocarbon Receptor Signaling				X
24	Assembly of RNA Polymerase II Complex				X
25	Assembly of RNA Polymerase III Complex				X
26	Atherosclerosis Signaling		X		
27	ATM Signaling				X
28	Autoimmune Thyroid Disease Signaling				X
29	Autophagy				X
30	Axonal Guidance Signaling				X
31	B Cell Activating Factor Signaling				X
32	B Cell Development				X
33	B Cell Receptor Signaling				X
34	BER pathway				X
35	BMP signaling pathway				X
36	Breast Cancer Regulation by Stathmin1				X
37	Bupropion Degradation	X			
38	Calcium Signaling				X
39	Calcium-induced T Lymphocyte Apoptosis				X
40	cAMP-mediated signaling	X	X		
41	Cancer Drug Resistance By Drug Efflux				X
42	Cardiac Hypertrophy Signaling				X
43	Cardiac β-adrenergic Signaling				X
44	Cardiomyocyte Differentiation via BMP Receptors	X			
45	Caveolar-mediated Endocytosis Signaling				X
46	CCR3 Signaling in Eosinophils				X
47	CCR5 Signaling in Macrophages				X
48	CD27 Signaling in Lymphocytes				X
49	CD28 Signaling in T Helper Cells				X
50	CD40 Signaling				X
51	Cdc42 Signaling				X
52	CDK5 Signaling				X
53	Cell Cycle Control of Chromosomal Replication				X
54	Cell Cycle Regulation by BTG Family Proteins				X
55	Cell Cycle: G1/S Checkpoint Regulation				X
56	Cell Cycle: G2/M DNA Damage Checkpoint Regulation				X
57	Ceramide Signaling				X
58	Chemokine Signaling				X
59	Cholecystokinin/Gastrin-mediated Signaling				X
60	Chronic Myeloid Leukemia Signaling				X
61	Clathrin-mediated Endocytosis Signaling				X
62	Cleavage and Polyadenylation of Pre-mRNA				X
63	CNTF Signaling				X
64	Coagulation System			X	
65	Colorectal Cancer Metastasis Signaling				X
66	Communication between Innate and Adaptive Immune Cells			X	
67	Corticotropin Releasing Hormone Signaling				X
68	CREB Signaling in Neurons				X
69	Crosstalk between Dendritic Cells and Natural Killer Cells			X	X
70	CTLA4 Signaling in Cytotoxic T Lymphocytes				X
71	CXCR4 Signaling				X
72	Cyclins and Cell Cycle Regulation				X
73	Cytotoxic T Lymphocyte-mediated Apoptosis of Target Cells				X
74	Death Receptor Signaling				X
75	Dendritic Cell Maturation				X
76	Dermatan Sulfate Degradation (Metazoa)			X	

77	Differential Regulation of Cytokine Production in Intestinal Epithelial Cells by IL-17A and IL-17F			X	
78	D-myo-inositol (1,4,5,6)-Tetrakisphosphate Biosynthesis			X	X
79	D-myo-inositol (3,4,5,6)-tetrakisphosphate Biosynthesis			X	X
80	D-myo-inositol-5-phosphate Metabolism			X	X
81	DNA damage-induced 14-3-3σ Signaling				X
82	DNA Double-Strand Break Repair by Homologous Recombination				X
83	DNA Double-Strand Break Repair by Non-Homologous End Joining				X
84	DNA Methylation and Transcriptional Repression Signaling				X
85	Dopamine-DARPP32 Feedback in cAMP Signaling				X
86	EGF Signaling				X
87	EIF2 Signaling	X			X
88	Endometrial Cancer Signaling				X
89	Endoplasmic Reticulum Stress Pathway				X
90	eNOS Signaling				X
91	Ephrin A Signaling				X
92	Ephrin B Signaling				X
93	Ephrin Receptor Signaling				X
94	Epithelial Adherens Junction Signaling				X
95	ErbB Signaling				X
96	ErbB2-ErbB3 Signaling				X
97	ErbB4 Signaling				X
98	ERK/MAPK Signaling				X
99	ERK5 Signaling				X
100	Erythropoietin Signaling				X
101	Estrogen Receptor Signaling				X
102	Estrogen-Dependent Breast Cancer Signaling				X
103	Estrogen-mediated S-phase Entry				X
104	Eumelanin Biosynthesis	X			
105	FAK Signaling				X
106	Fc Epsilon RI Signaling				X
107	Fcγ Receptor-mediated Phagocytosis in Macrophages and Monocytes				X
108	FcγRIIB Signaling in B Lymphocytes				X
109	FGF Signaling				X
110	FLT3 Signaling in Hematopoietic Progenitor Cells				X
111	fMLP Signaling in Neutrophils				X
112	FXR/RXR Activation		X		
113	G Beta Gamma Signaling				X
114	GABA Receptor Signaling		X		
115	GABA Receptor Signaling			X	
116	GADD45 Signaling				X
117	Gap Junction Signaling				X
118	GDNF Family Ligand-Receptor Interactions				X
119	Germ Cell-Sertoli Cell Junction Signaling				X
120	Glioblastoma Multiforme Signaling				X
121	Glioma Invasiveness Signaling			X	X
122	Glioma Signaling				X
123	Glucocorticoid Receptor Signaling				X
124	Glutamate Receptor Signaling	X	X		
125	GM-CSF Signaling				X
126	GNRH Signaling				X
127	G-Protein Coupled Receptor Signaling		X		X
128	Graft-versus-Host Disease Signaling				X
129	Granulocyte Adhesion and Diapedesis	X		X	
130	Granzyme A Signaling				X
131	Granzyme B Signaling				X
132	Growth Hormone Signaling				X
133	Gustation Pathway	X		X	
134	Gα12/13 Signaling				X
135	Gαi Signaling	X	X		
136	Gαq Signaling				X
137	Gαs Signaling		X		
138	Hepatic Fibrosis / Hepatic Stellate Cell Activation		X	X	
139	HER-2 Signaling in Breast Cancer				X
140	Hereditary Breast Cancer Signaling				X
141	HGF Signaling				X
142	HIF1α Signaling				X
143	HIPPO signaling				X
144	HMGB1 Signaling			X	X
145	Huntington's Disease Signaling				X
146	Hypoxia Signaling in the Cardiovascular System				X
147	iCOS-iCOSL Signaling in T Helper Cells				X
148	IGF-1 Signaling				X
149	IL-1 Signaling				X
150	IL-10 Signaling				X
151	IL-12 Signaling and Production in Macrophages				X
152	IL-15 Production				X
153	IL-15 Signaling				X
154	IL-17 Signaling				X
155	IL-17A Signaling in Airway Cells				X

156	IL-17A Signaling in Fibroblasts				X
157	IL-2 Signaling				X
158	IL-22 Signaling				X
159	IL-3 Signaling				X
160	IL-4 Signaling				X
161	IL-6 Signaling				X
162	IL-8 Signaling				X
163	IL-9 Signaling				X
164	ILK Signaling				X
165	Induction of Apoptosis by HIV1				X
166	Inhibition of Angiogenesis by TSP1				X
167	Inhibition of Matrix Metalloproteases				X
168	iNOS Signaling			X	
169	Insulin Receptor Signaling				X
170	Integrin Signaling				X
171	Interferon Signaling				X
172	JAK/Stat Signaling				X
173	Leptin Signaling in Obesity				X
174	Leukocyte Extravasation Signaling				X
175	Lipid Antigen Presentation by CD1				X
176	LPS/IL-1 Mediated Inhibition of RXR Function		X		
177	LPS-stimulated MAPK Signaling				X
178	LXR/RXR Activation		X		
179	Lymphotoxin β Receptor Signaling				X
180	Macropinocytosis Signaling				X
181	Mechanisms of Viral Exit from Host Cells				X
182	Melanocyte Development and Pigmentation Signaling				X
183	Melanoma Signaling				X
184	Melatonin Degradation I	X	X		X
185	MIF Regulation of Innate Immunity				X
186	MIF-mediated Glucocorticoid Regulation				X
187	Mismatch Repair in Eukaryotes				X
188	Mitochondrial Dysfunction	X			X
189	Mitotic Roles of Polo-Like Kinase				X
190	Molecular Mechanisms of Cancer				X
191	Mouse Embryonic Stem Cell Pluripotency				X
192	mTOR Signaling				X
193	Myc Mediated Apoptosis Signaling				X
194	Natural Killer Cell Signaling				X
195	Neuregulin Signaling				X
196	Neuropathic Pain Signaling In Dorsal Horn Neurons		X		X
197	Neurotrophin/TRK Signaling				X
198	NF-κB Activation by Viruses				X
199	NF-κB Signaling				X
200	NGF Signaling				X
201	Nicotine Degradation II	X	X		
202	Nicotine Degradation III	X	X		
203	nNOS Signaling in Skeletal Muscle Cells			X	
204	Non-Small Cell Lung Cancer Signaling				X
205	NRF2-mediated Oxidative Stress Response				X
206	Nucleotide Excision Repair Pathway				X
207	Nur77 Signaling in T Lymphocytes				X
208	Oncostatin M Signaling				X
209	Ovarian Cancer Signaling				X
210	Oxidative Phosphorylation	X			X
211	P2Y Purigenic Receptor Signaling Pathway				X
212	p38 MAPK Signaling				X
213	p53 Signaling				X
214	p70S6K Signaling				X
215	PAK Signaling				X
216	Pancreatic Adenocarcinoma Signaling				X
217	Paxillin Signaling				X
218	PDGF Signaling				X
219	PEDF Signaling				X
220	phagosome formation				X
221	phagosome maturation				X
222	Phospholipase C Signaling				X
223	PI3K Signaling in B Lymphocytes				X
224	PI3K/AKT Signaling				X
225	PKCθ Signaling in T Lymphocytes				X
226	Polyamine Regulation in Colon Cancer				X
227	PPAR Signaling				X
228	PPARα/RXRα Activation				X
229	Primary Immunodeficiency Signaling				X
230	Production of Nitric Oxide and Reactive Oxygen Species in Macrophages			X	X
231	Prolactin Signaling				X
232	Prostate Cancer Signaling				X
233	Protein Kinase A Signaling				X
234	Protein Ubiquitination Pathway				X

235	PTEN Signaling				X
236	Pyridoxal 5'-phosphate Salvage Pathway				X
237	Rac Signaling				X
238	RAN Signaling				X
239	RANK Signaling in Osteoclasts				X
240	RAR Activation				X
241	Reelin Signaling in Neurons				X
242	Regulation of Actin-based Motility by Rho				X
243	Regulation of Cellular Mechanics by Calpain Protease				X
244	Regulation of eIF4 and p70S6K Signaling				X
245	Regulation of IL-2 Expression in Activated and Anergic T Lymphocytes				X
246	Regulation of the Epithelial-Mesenchymal Transition Pathway				X
247	Relaxin Signaling				X
248	Remodeling of Epithelial Adherens Junctions				X
249	Renal Cell Carcinoma Signaling				X
250	Renin-Angiotensin Signaling				X
251	Retinoic acid Mediated Apoptosis Signaling				X
252	RhoGDI Signaling				X
253	Role of BRCA1 in DNA Damage Response				X
254	Role of CHK Proteins in Cell Cycle Checkpoint Control				X
255	Role of IL-17A in Arthritis				X
256	Role of JAK family kinases in IL-6-type Cytokine Signaling				X
257	Role of JAK1 and JAK3 in γc Cytokine Signaling				X
258	Role of JAK1, JAK2 and TYK2 in Interferon Signaling				X
259	Role of Macrophages, Fibroblasts and Endothelial Cells in Rheumatoid Arthritis				X
260	Role of MAPK Signaling in the Pathogenesis of Influenza				X
261	Role of NANOG in Mammalian Embryonic Stem Cell Pluripotency				X
262	Role of NFAT in Cardiac Hypertrophy				X
263	Role of NFAT in Regulation of the Immune Response				X
264	Role of Oct4 in Mammalian Embryonic Stem Cell Pluripotency				X
265	Role of p14/p19ARF in Tumor Suppression				X
266	Role of PI3K/AKT Signaling in the Pathogenesis of Influenza				X
267	Role of PKR in Interferon Induction and Antiviral Response				X
268	Role of RIG1-like Receptors in Antiviral Innate Immunity				X
269	Role of Tissue Factor in Cancer				X
270	Salvage Pathways of Pyrimidine Ribonucleotides				X
271	SAPK/JNK Signaling				X
272	Semaphorin Signaling in Neurons			X	X
273	Signaling by Rho Family GTPases				X
274	Small Cell Lung Cancer Signaling				X
275	Sperm Motility		X		
276	Sphingosine-1-phosphate Signaling				X
277	STAT3 Pathway				X
278	Superpathway of Inositol Phosphate Compounds				X
279	Superpathway of Melatonin Degradation	X			
280	Synaptic Long Term Depression		X		X
281	Synaptic Long Term Potentiation				X
282	Systemic Lupus Erythematosus Signaling				X
283	T Cell Receptor Signaling				X
284	T Helper Cell Differentiation				X
285	Tec Kinase Signaling				X
286	Telomerase Signaling				X
287	Telomere Extension by Telomerase				X
288	TGF-β Signaling			X	X
289	Thrombin Signaling				X
290	Thrombopoietin Signaling				X
291	Tight Junction Signaling				X
292	TNFR1 Signaling				X
293	TNFR2 Signaling				X
294	Transcriptional Regulatory Network in Embryonic Stem Cells		X		
295	TREM1 Signaling				X
296	Tumoricidal Function of Hepatic Natural Killer Cells				X
297	TWEAK Signaling				X
298	Type I Diabetes Mellitus Signaling				X
299	Type II Diabetes Mellitus Signaling				X
300	Unfolded protein response			X	
301	Unfolded protein response				X
302	UVA-Induced MAPK Signaling				X
303	UVB-Induced MAPK Signaling				X
304	UVC-Induced MAPK Signaling				X
305	VEGF Family Ligand-Receptor Interactions				X
306	VEGF Signaling				X
307	Virus Entry via Endocytic Pathways				X
308	Wnt/β-catenin Signaling				X
309	Xenobiotic Metabolism Signaling				X
310	α-Adrenergic Signaling				X
