# Supplementary material for: Sm-p80-based schistosomiasis vaccine mediated epistatic interactions identified potential immune signatures for vaccine efficacy in mice and baboons
Source: PLoS One. 2017 Feb 13;12(2):e0171677. doi: 10.1371/journal.pone.0171677 (PMC5305113; doi:10.1371/journal.pone.0171677)
Supplement: S2 Fig — CIRCOS plot for each time-point examined (p<0.01 and >2-fold change) during the developing immune response, the colored bar on the outermost layer represents the total number of DEGs with gene names. Blue indicates 24 hours, yellow 48 hours, red 7 days, and green 21 days. Inner layer depicts fold change of each corresponding gene, where red is up-regulation and blue is down-regulation. Inter-connecting links represent DE transcripts that are shared between time points examined; where green indicates overlaps between 24 hours and 48 hours; orange for 48 hour and 7 days; yellow for 7 days and 21 days; aqua for 21 days and 24 hours; violet for 24 hours and 7 days; and light-green for 48 hours and 21 days. (PDF) [file pone.0171677.s009.pdf]

S2 Fig

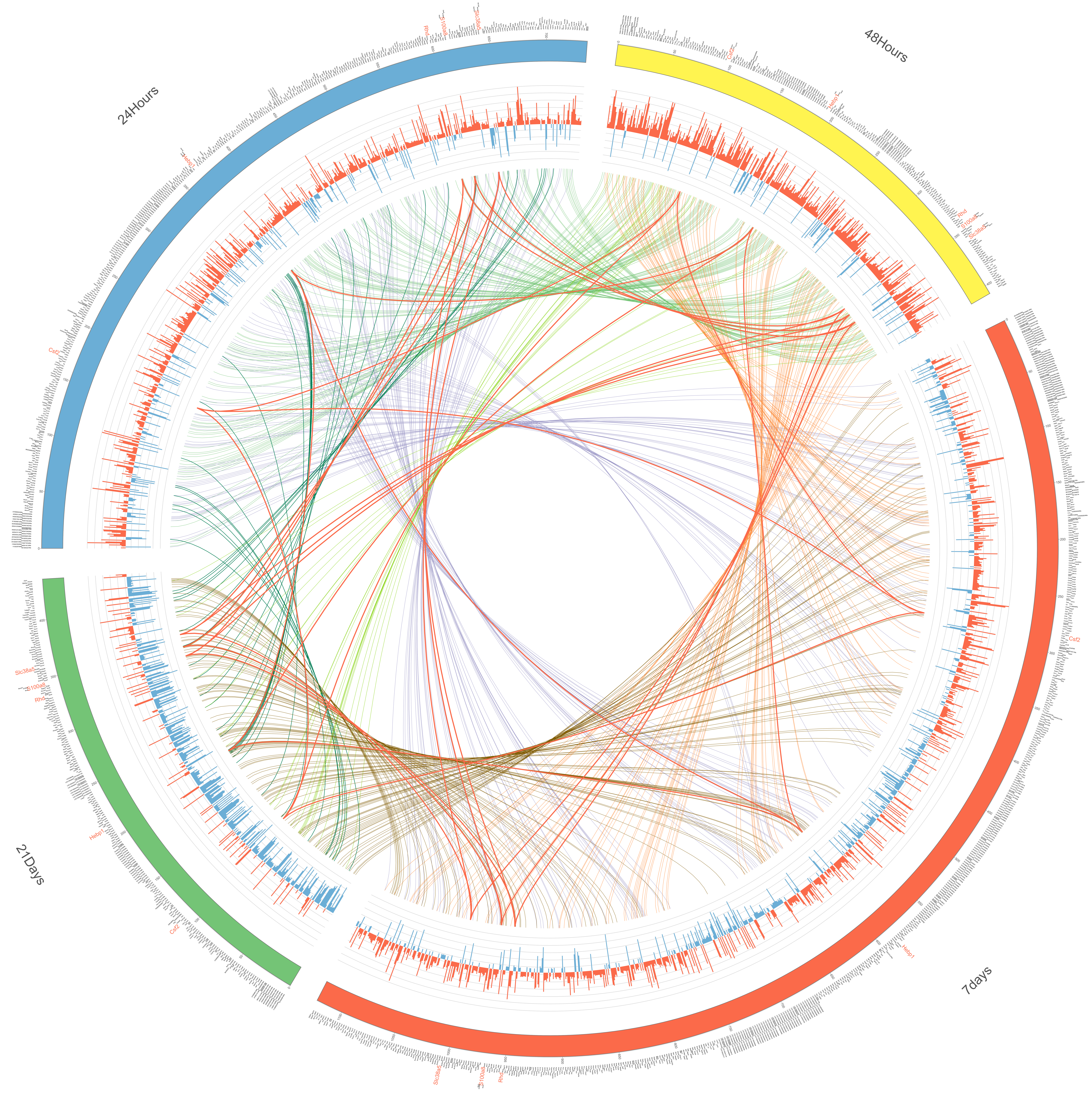
24Hours
48Hours
7days
21Days
Rhd
S100a8
Slc38a5
Hebp1
Csf2
