# Supplementary material for: Sm-p80-based schistosomiasis vaccine mediated epistatic interactions identified potential immune signatures for vaccine efficacy in mice and baboons
Source: PLoS One. 2017 Feb 13;12(2):e0171677. doi: 10.1371/journal.pone.0171677 (PMC5305113; doi:10.1371/journal.pone.0171677)

S6 Fig

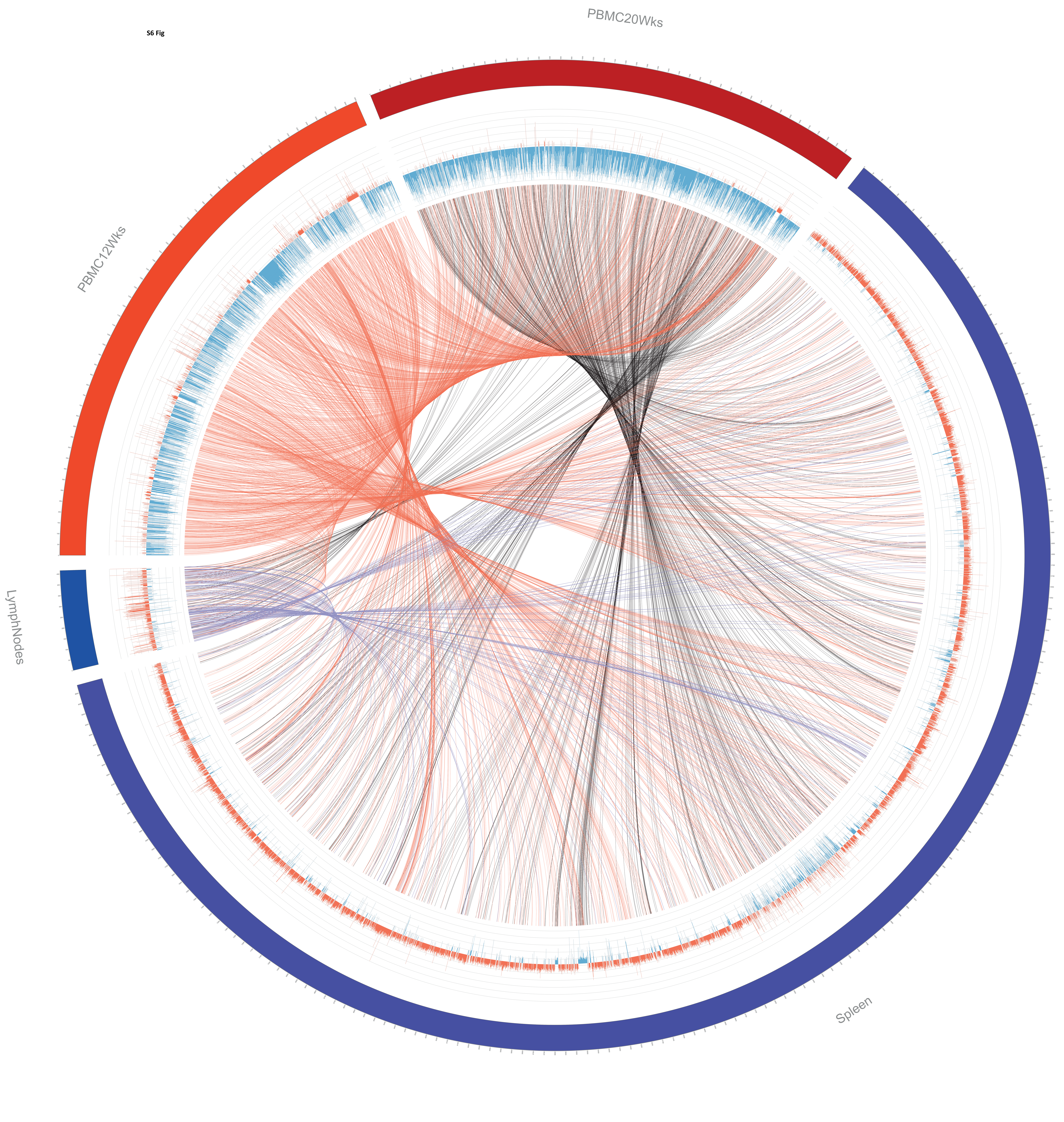

Supplement: S6 Fig — Circular visualization, CIRCOS, plot of statistically significant (p<0.1 and >2-fold change) differentially expressed genes from vaccinated baboons’ PBMCs at week 12 (red), PBMCs at week 20 (burgundy), spleen (purple), and lymph nodes (blue). Inner layer depicts fold change of each corresponding gene, where red is upregulation and blue is downregulation. Links indicate the transcripts that are shared between tissues. (PDF) [file pone.0171677.s013.pdf]
